# Inhibition of SRP-dependent protein secretion by the bacterial alarmone (p)ppGpp

**DOI:** 10.1038/s41467-022-28675-0

**Published:** 2022-02-25

**Authors:** Laura Czech, Christopher-Nils Mais, Hanna Kratzat, Pinku Sarmah, Pietro Giammarinaro, Sven-Andreas Freibert, Hanna Folke Esser, Joanna Musial, Otto Berninghausen, Wieland Steinchen, Roland Beckmann, Hans-Georg Koch, Gert Bange

**Affiliations:** 1 Center for Synthetic Microbiology (SYNMIKRO) and Department of Chemistry Philipps-Universität Marburg, Marburg, Germany; 2 Gene Center Munich, Department of Biochemistry, Ludwig-Maximilians-Universität, LMU, Munich, Germany; 3 Institute of Biochemistry and Molecular Biology, Faculty of Medicine, Albert-Ludwigs-Universität Freiburg, Freiburg, Germany; 4 Faculty of Biology, Albert-Ludwigs-Universität Freiburg, Freiburg, Germany; 5 Institut für Zytobiologie, Philipps-Universität Marburg, Marburg, Germany; 6 Core Facility “Protein Biochemistry and Spectroscopy”, Philipps-Universität Marburg, Marburg, Germany; 7 Max-Planck Institute for terrestrial Microbiology, Marburg, Germany

**Keywords:** Bacterial secretion, Cryoelectron microscopy, Bacterial structural biology, Nucleotide-binding proteins

## Abstract

The stringent response enables bacteria to respond to nutrient limitation and other stress conditions through production of the nucleotide-based second messengers ppGpp and pppGpp, collectively known as (p)ppGpp. Here, we report that (p)ppGpp inhibits the signal recognition particle (SRP)-dependent protein targeting pathway, which is essential for membrane protein biogenesis and protein secretion. More specifically, (p)ppGpp binds to the SRP GTPases Ffh and FtsY, and inhibits the formation of the SRP receptor-targeting complex, which is central for the coordinated binding of the translating ribosome to the SecYEG translocon. Cryo-EM analysis of SRP bound to translating ribosomes suggests that (p)ppGpp may induce a distinct conformational stabilization of the NG domain of Ffh and FtsY in *Bacillus subtilis* but not in *E. coli*.

## Abstract (web summary)

Bacterial responses to nutrient limitation and other stress conditions are often modulated by the nucleotide-based second messenger (p)ppGpp. Here, the authors show that (p)ppGpp inhibits the SRP membrane-protein insertion and secretion pathway by binding to GTPases Ffh and FtsY.

## Introduction

Central to the bacterial response to starvation and stress are the guanosine-based second messengers ppGpp and pppGpp (also: (p)ppGpp or “alarmones”)^1,2^. Biosynthesis and degradation of (p)ppGpp relies on RelA/SpoT homology (RSH)-type proteins^3,4^. Most prominent and conserved are the RelA/Rel proteins, which sense amino acid starvation during the stringent response by detecting ribosomes blocked by cognate, uncharged tRNAs at their A-site^5–9^. The (p)ppGpp synthetase activity of these enzymes is stimulated upon binding to stalled ribosomes by a molecular mechanism conserved in Gram-negative and -positive bacteria^9–12^. Besides the Rel/RelA enzymes, many bacterial species contain further RSH-enzymes commonly referred to as small alarmone synthetases and hydrolases (summarized in ref. ^4^).

As the most prominent consequence of elevated alarmone levels, protein biosynthesis is downregulated through direct inhibition of translational GTPases (recently summarized in refs. ^2,13^). Yet, alarmones effect a wide range of physiological and metabolic processes by their specific interactions with numerous proteins and also RNA targets (recently summarized in refs. ^14,15^). A recent affinity-based screening approach for (p)ppGpp targets in *Escherichia coli* supported this idea by identifying over 50 potential targets^16^. Notably, the bacterial signal recognition particle (SRP) GTPases Ffh and FtsY were also identified in this screen, suggesting that (p)ppGpp may potentially also regulate the essential SRP-dependent pathway of membrane protein insertion. This appears plausible, since in bacteria the biogenesis of a large portion of transmembrane and some secreted proteins relies on the SRP machinery (reviewed in refs. ^17–20^). However, this idea has never been challenged and thus a mechanistic understanding of how (p)ppGpp could modulate the SRP pathway is not available to date.

SRP is a conserved ribonucleoprotein particle consisting of the GTPase Ffh and the SRP-RNA^20–22^ and its mode of operation is well understood^23–26^. Briefly, SRP recognizes hydrophobic signal sequences in the context of translating ribosomes, and SRP bound to ribosome-nascent chain complexes (RNCs) in turn interacts in a GTP-dependent manner with its receptor FtsY, also a GTPase, which localizes at the cytoplasmic membrane periphery of bacteria via a membrane-targeting sequence^27,28^. Consequently, the RNC is transferred onto the SecYEG translocon followed by dissociation of the SRP-FtsY complex after hydrolysis of GTP enabling the initiation of a new round of SRP-mediated protein targeting. Ffh and FtsY are multi-domain proteins, which share the highly homologous NG domain (reviewed in ref. ^20^). The NG domain consists of a bundle of four α-helices followed by a GTPase (G) domain common to small G proteins, such as Ras^29,30^. When bound to GTP, the NG domains of Ffh and FtsY form the heterodimeric targeting complex, which regulates the transfer of a RNC to a vacant and membrane-embedded translocon through a series of conformational rearrangements (summarized in ref. ^21^). Within the targeting complex, both GTPases share a composite active site in which two GTPs reciprocally align, such that the 3′-OH group of the ribose of one GTP interacts with the γ-phosphate of the other, and vice versa^31–33^. This unique nucleotide arrangement is essential for productive formation of the SRP-FtsY complex^31^, and the reciprocal stimulation of both GTPase activities in the context of the SRP-RNA^34^, which finally enables productive transfer of the RNC onto the translocon. However, a widely unaddressed question is whether the SRP-mediated protein-targeting pathway is subject to regulation in response to stress conditions, e.g., amino acid starvation.

Here, we show that the alarmones (p)ppGpp specifically bind to the GTPase domains of SRP and its receptor FtsY resulting in an inhibition of targeting complex formation. This in turn restricts the SRP-dependent pathway of membrane protein insertion and secretion during stressful times.

## Results

### In vitro inhibition of SRP-dependent post-translational membrane protein targeting and insertion by (p)ppGpp

To investigate whether and how the alarmones (p)ppGpp would interfere with the SRP-dependent targeting process, we analyzed the influence of ppGpp and pppGpp on the SRP-dependent insertion of membrane proteins. As a first model, we chose the single-spanning membrane protein YohP from *E. coli*, which was recently shown to be strictly dependent on SRP/FtsY for membrane insertion^18,35^. A major advantage of using YohP is that SRP acts post-translationally during YohP insertion and thus the (p)ppGpp effect on SRP/FtsY-dependent insertion can be analyzed without impairing the GTP-dependent steps of translation, which are also known targets of (p)ppGpp (recently summarized in refs. ^2,14,15^).

**Fig. 1:**
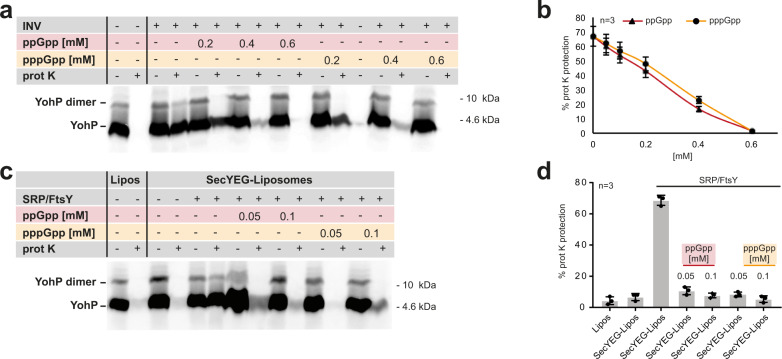
Post-translational targeting of SRP substrate YohP is inhibited by (p)ppGpp. **a** YohP was in vitro synthesized using a purified coupled transcription/translation system (CTF system) and translation was terminated by the addition of chloramphenicol (35 mg mL^−1^). Samples were then centrifuged for removing ribosomes and aggregates and the supernatant was incubated with INV (inner membrane vesicles) or INV-buffer for 10 min in the presence of 10 µM GTP. Where indicated, ppGpp or pppGpp were added together with INV. Subsequently, one half of the sample was directly TCA precipitated, while the other half was first treated with proteinase K (prot K) before TCA precipitation. Samples were then separated by SDS-PAGE and analyzed by autoradiography. **b** Quantification of three independent experiments as described in **a** and the mean values (±SD) are shown. **c** As in **a**, but insertion was analyzed in the presence of liposomes of reconstituted SecYEG-proteoliposomes. Liposomes were generated from *E. coli* phospholipids and contained 70% PE, 25% PG and 5% CL, and proteoliposomes contained 100 ng µL^−1^ SecYEG complex. When indicated, insertion was performed in the presence of absence of purified SRP/FtsY (20 ng µL^−1^, each) and at the indicated (p)ppGpp concentrations. Uncropped images are shown in the Supplementary Material (Supplementary Fig. 2). Samples were further processed as described in **a**. **d** Quantification of three independent experiments as described in **c** and the mean values (±SD) are shown.

YohP was in vitro synthesized and ^35^S-labeled using an *E. coli* coupled transcription/translation system^36^. Translation was subsequently terminated by the addition of chloramphenicol and ribosomes were removed by ultracentrifugation. In vitro synthesized YohP was then incubated with *E. coli* inverted inner membrane vesicles (INV) and membrane insertion was determined by proteinase K (prot. K) resistance. In the presence of INV, almost 70% of the in vitro synthesized YohP were prot. K resistant, indicating that insertion into the *E. coli* membrane had occurred (Fig. 1a, b). In contrast, basically all YohP was degraded in the absence of INV. When YohP was incubated with INV in the presence of increasing ppGpp or pppGpp levels, a concentration-dependent decrease of YohP insertion was observed for both ppGpp and pppGpp (Fig. 1a, b), with a complete block of insertion at 0.6 mM (p)ppGpp. These data demonstrate that both ppGpp and pppGpp inhibit membrane insertion of the SRP-dependent membrane protein YohP.

The assays described above do not require the addition of purified SRP/FtsY, because sufficient amounts of both proteins are bound to INV^36^. This, however, makes it difficult to assign the observed inhibition of YohP insertion by (p)ppGpp to impaired SRP/FtsY activity. Therefore, we assayed the effect of (p)ppGpp on YohP insertion in a highly purified assay system, which consisted of reconstituted SecYEG-proteoliposomes, and purified SRP and FtsY. When in vitro synthesized YohP was incubated with liposomes or SecYEG-proteoliposomes without adding SRP/FtsY, no significant prot. K resistance was observed (Fig. 1c, d). However, in the presence of SRP/FtsY and SecYEG-proteoliposomes about 70% of the in vitro synthesized YohP was resistant against prot. K treatment, indicative for membrane insertion (Fig. 1c, d). No prot. K protection of YohP was observed when SRP/FtsY were added to liposomes or in the presence of just SRP/FtsY (Supplementary Fig. 1). Adding ppGpp or pppGpp together with SRP/FtsY reduced YohP insertion into SecYEG proteoliposomes drastically, further validating that ppGpp and pppGpp inhibit SRP and FtsY and thus block YohP insertion. The (p)ppGpp concentrations required for inhibiting YohP insertion into SecYEG-proteoliposomes were much lower than the concentrations required for inhibition in the INV system (Fig. 1c, d). This likely reflects the fact that in addition to SRP and FtsY, additional GTP-binding proteins are present in INV, which may also bind (p)ppGpp. Taken together, these experiments clearly show that (p)ppGpp efficiently inhibits the SRP-dependent membrane insertion of membrane proteins via the SecYEG translocon.

### In vitro inhibition of SRP-dependent co-translational membrane protein targeting and insertion by (p)ppGpp

**Fig. 2:**
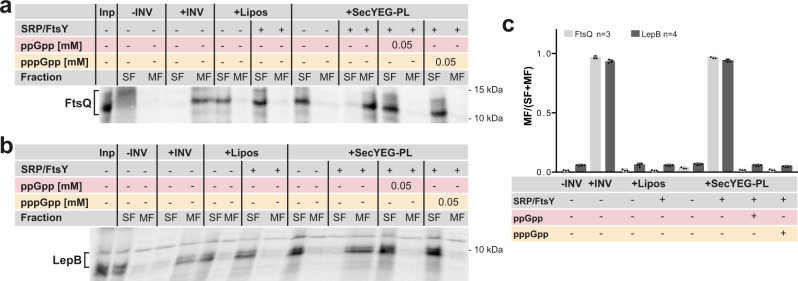
Co-translational membrane targeting of ribosome-nascent chains (RNCs) is inhibited by (p)ppGpp. **a** and **b** RNCs of the SRP substrates FtsQ (**a**) or LepB (**b**) were in vitro synthesized (input, Inp) and incubated with INV (inner membrane vesicles) or reconstituted SecYEG proteoliposomes (SecYEG-PL; 100 ng SecYEG µL^−1^). INV buffer and liposomes (lipos) served as a control. When indicated, purified SRP and FtsY (20 ng µL^−1^ each) and ppGpp or pppGpp (50 µM final concentration) were present during incubation. Samples were then subjected to floatation gradient centrifugation and the membrane fraction (MF) and soluble fraction (SF) were were separated and analyzed by SDS-PAGE and autoradiography. Uncropped images are shown in the Supplementary Material (Supplementary Fig. 2) **c** Quantification of membrane targeting of FtsQ-RNCs (*n* = 3, biologically independent experiments) and LepB-RNCs (*n* = 4, biologically independent experiments) in the presence of (p)ppGpp. Radioactively labeled bands in the MF and SF were quantified using the *ImageQuant* software and are displayed as MF/(SF + MF). Error bars indicate the SEM values, which were determined using GraphPad Prism.

Since SRP-dependent targeting of YohP occurs post-translationally and thus deviates from the canonical co-translational SRP-dependent targeting, we also tested the influence of (p)ppGpp on co-translational membrane targeting of LepB- and FtsQ-RNCs, which are classical SRP substrates^18,37,38^. LepB- and FtsQ-RNCs were in vitro synthesized and incubated with INV or SecYEG-proteoliposomes in the presence or absence of SRP/FtsY and (p)ppGpp. Efficient membrane targeting of these RNCs was then analyzed by floatation analyses, which separates membrane-bound RNCs from non-bound RNCs^39^. In the presence of INV, both RNCs were almost exclusively found in the membrane fraction (MF) but remained in the soluble fraction in the absence of INV or in the presence of liposomes (Fig. 2a, b). When membrane binding of LepB- and FtsQ-RNCs to SecYEG-proteoliposomes was analyzed, both RNCs were found in the membrane fraction when purified SRP and FtsY were present but were recovered from the soluble fraction in their absence (Fig. 2a, b). This demonstrates that the assay reliably reports co-translational targeting of RNCs to the SecYEG-translocon by SRP and its receptor FtsY. Importantly, the addition of either ppGpp or pppGpp completely blocked membrane targeting of LepB- and FtsQ-RNCs (Fig. 2a, b). Quantification of several (*n* ≥ 3) independent experiments confirmed the inhibitory effect of (p)ppGpp on SRP-dependent targeting of RNCs to the SecYEG-translocon (Fig. 2c). In summary, these data demonstrate that (p)ppGpp inhibit also co-translational membrane targeting by the SRP pathway.

### (p)ppGpp reduces GTPase activities of SRP and its receptor

**Fig. 3:**
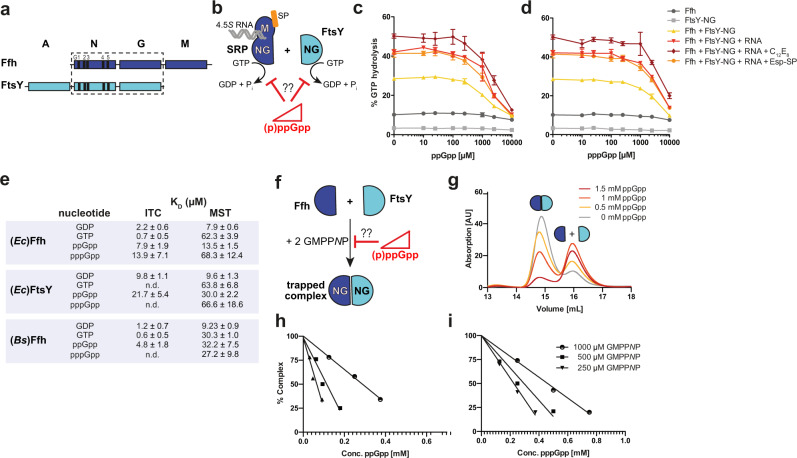
(p)ppGpp reduces GTPase activity and complex formation of SRP and FtsY. **a** Domain architecture of the bacterial SRP-GTPases Ffh (blue) and FtsY (cyan), both sharing the conserved GTPase-containing NG domain. The G-elements G1–G5 as well as the A and M domains specific to FtsY and Ffh, respectively, are shown. **b** Scheme of the experimental setup for analyzing the impact of increasing concentrations of (p)ppGpp on the GTPase activities of SRP and FtsY. Orange sphere depicts the signal peptide (SP), and gray strands the SRP RNA. **c** and **d** GTPase activity of full-length Ffh and FtsY-NG was assayed in the presence of increasing amounts of the competitors ppGpp (**c**) and pppGpp (**d**). Where indicated, 5 µM (*Ec*)FtsY-NG, 6 µM of 4.5S RNA, 5 µM Esp-signal peptide and 100 µM C_12_E_8_ (signal peptide mimic) were added to the reaction including 5 µM full-length (*Ec*)Ffh and 1 mM GTP. The data represent mean values (±SD) of *n* = 3 replicates. **e** The table lists the *K*_D_ values obtained for the binding of GDP, GTP, ppGpp and pppGpp to (*Ec*)Ffh, (*Ec*)FtsY- and (*Bs*)Ffh-NG domains determined either by isothermal titration calorimetry (ITC) or microscale thermophoresis (MST). **f** Scheme of the experimental setup for analyzing the impact of increasing concentrations of (p)ppGpp on the GTP-dependent formation of the Ffh/FtsY-NG domain complex. **g** Analytical size-exclusion chromatography (SEC) monitoring the complex formation and dissociation of Ffh-NG and FtsY-NG (100 µM each) incubated with 1 mM GMPP*N*P and in the absence or presence of increasing ppGpp concentrations (0, 0.5, 1, and 1.5 mM). **h** and **i** Percentage of formed Ffh-NG/FtsY-NG complexes (50 µM each) in the presence of different GMPP*N*P concentrations (250, 500, and 1,000 µM) analyzed in the presence of increasing ppGpp (**h**) and pppGpp (**i**) concentrations, respectively.

SRP/FtsY complex formation followed by the subsequent GTPase activities of Ffh and FtsY is required for the successful delivery of target proteins to the SecYEG translocon^18,35^. Therefore, we tested whether GTP hydrolysis would be impaired in the presence of (p)ppGpp. Hence, full-length (*Ec*)Ffh and the NG domain of (*Ec*)FtsY (Fig. 3a) were tested for their GTP hydrolysis activity in the presence of 4.5S RNA (i.e., the SRP-RNA), the well characterized ΔEspP signal peptide and the signal peptide mimicking detergent C_12_E_8_ (nonionic detergent octaethyleneglycol dodecylether), which all have been shown to stimulate the GTPase activity of the targeting complex (Fig. 3b)^40^. The assays were conducted using 1 mM GTP and increasing amounts of ppGpp (Fig. 3c) and pppGpp (Fig. 3d) ranging from 0 to 10 mM. While the GTPase activities of full-length (*Ec*)Ffh and (*Ec*)FtsY-NG alone were very low, GTP hydrolysis was stimulated when both proteins were present (Fig. 3c, d)^41^. The GTPase activities were further stimulated in the presence of 4.5S RNA^41,42^, and through the addition of the ΔEspP signal peptide or the signal peptide mimic C_12_E_8_ (Fig. 3c, d)^40^. In each of the tested conditions, addition of (p)ppGpp resulted in a reduction of GTP hydrolysis when the alarmones were supplemented at concentrations equimolar to the GTP substrate (1 mM) or higher consistent with a competitive mode of inhibition, whereby ppGpp appeared to be a slightly more potent inhibitor than pppGpp (Fig. 3c, d). Taken together, our data show that the alarmones (p)ppGpp inhibit the GTPase activity observed when SRP and its receptor are present, irrespective of the presence or absence of the SRP-RNA, the signal peptide or its mimicry. It is well established that (p)ppGpp levels rise approximately three times above the GTP levels under stringent conditions in the model organisms *E. coli*^43–45^ and *B. subtilis*^46–48^, further supporting the physiological relevance of the (p)ppGpp action on the SRP machinery.

### Binding of (p)ppGpp to the NG domains of Ffh and its receptor FtsY

Our data suggested that (p)ppGpp directly interferes with the GTPases of SRP and its receptor FtsY (Fig. 3a–d). To understand the action of (p)ppGpp on Ffh and FtsY GTPases further mechanistically, we next employed the GTPase-containing NG domains of *E. coli* (*Ec*) Ffh and FtsY (Ffh-NG and FtsY-NG, respectively; Fig. 3a) and probed their binding affinity for ppGpp and pppGpp by isothermal titration calorimetry (ITC). Determination of the dissociation constants (*K*_D_) of (*Ec*)Ffh-NG revealed an affinity of 7.9 ± 1.9 µM for ppGpp-binding while its counterpart GDP exhibited an affinity of 2.2 ± 0.6 µM (Fig. 3e; Supplementary Figs. 3 and 4). For the NG domain of the SRP-receptor (*Ec*)FtsY a *K*_D_ of 21.7 ± 5.4 µM for ppGpp and 9.8 ± 1.1 µM for GDP were obtained (Fig. 3e and Supplementary Figs. 3 and 4). These *K*_D_ values show that ppGpp exhibits similar binding affinities as its GDP counterpart for the SRP-GTPases Ffh and FtsY.

Next, we wanted to probe the affinities of the Ffh and FtsY-NG-domains for GTP and its counterpart pppGpp. Determination of *K*_D_ by ITC of (*Ec*)Ffh-NG revealed an affinity of 13.9 ± 7.1 µM for pppGpp-binding while its counterpart GTP exhibited an affinity of 0.7 ± 0.5 µM (Fig. 3e; Supplementary Figs. 3 and 4). For the (*Ec*)FtsY-NG, we were unable to determine a reliable *K*_D_-value by ITC, because of protein aggregation in the presence of either of the two nucleotides during the ITC runs. It might be that the exposure of FtsY-NG to either GTP or pppGpp leads to an expelling of the amphipathic membrane-targeting sequence (MTS)^27,28^, thus causing the observed aggregation under the relatively high protein concentrations (i.e., 50 µM) used for the ITC experiments. Thus, we decided to recapitulate the *K*_D_’s of (*Ec*)Ffh-NG and (*Ec*)FtsY-NG for ppGpp and pppGpp by microscale thermophoresis (MST), which requires much less protein concentration. Our MST experiments show that the alarmones pppGpp and ppGpp bind with similar *K*_D_’s as their GTP and GDP counterparts, respectively, to either (*Ec*)Ffh-NG and (*Ec*)FtsY-NG (Fig. 3e and Supplementary Fig. 4). We would like to note that the *K*_D_-values for GTP and pppGpp measured by MST are somewhat higher than those obtainable by ITC (Fig. 3e) and other methods (e.g., refs. ^41,49^). The reason for this observation is likely due to required labeling of the analyzed proteins with the fluorescent dye at lysine residues, which might weaken the binding of nucleotides. Despite of this, (*Ec*)Ffh-NG and (*Ec*)FtsY-NG show similar *K*_D_-values for GTP and pppGpp when measured by MST (Fig. 3e; Supplementary Figs. 3 and 4). This strongly suggests that pppGpp exhibits similar binding affinities as its GTP counterpart for the SRP-GTPases Ffh and FtsY. Overall, these observations indicate that the alarmones can act as competitive inhibitors of GDP/GTP by occupying the same binding sites in FtsY and Ffh. Our data might also suggest that SRP-GTPases slightly favor the binding of ppGpp over pppGpp.

### Disruption of Ffh-FtsY complex formation in the presence of (p)ppGpp

If (p)ppGpp can indeed act as a competitive inhibitor of the GTPases SRP/FtsY, we asked whether they would directly interfere with the strictly GTP-dependent formation of the SRP/FtsY-targeting complex (Fig. 3f). Thus, we incubated equal amounts of (*Ec*)Ffh-NG and (*Ec*)FtsY-NG in the presence of the non-hydrolysable GTP analog guanosine-5′-[(β,γ)-imido]triphosphate (GMPP*N*P), which enabled trapping of the *E. coli* Ffh/FtsY heterodimer stabilized by two GTP-mimicking GMPP*N*P molecules, followed by quantitative analysis of the complex formation by SEC (Fig. 3f, g). We next analyzed to which extent the presence of either ppGpp or pppGpp affects GMPP*N*P-dependent Ffh/FtsY complex formation (Fig. 3g–i). Thus, we added increasing amounts of each of the two alarmones and analyzed the complex formation in the presence of either 0.25, 0.5 or 1 mM GMPP*N*P. These experiments show that complex formation was half-inhibited at equal (p)ppGpp/GMPP*N*P ratios and roughly abolished when the (p)ppGpp concentrations exceeded those of GMPP*N*P by two-fold (Fig. 3h, i for ppGpp and pppGpp, respectively). These data further consolidate the idea that (p)ppGpp acts as competitive inhibitor of GTP, because formation of the Ffh/FtsY-targeting complex requires two GTP (or GMPP*N*P) molecules.

### Crystallographic analysis of alarmone binding

**Fig. 4:**
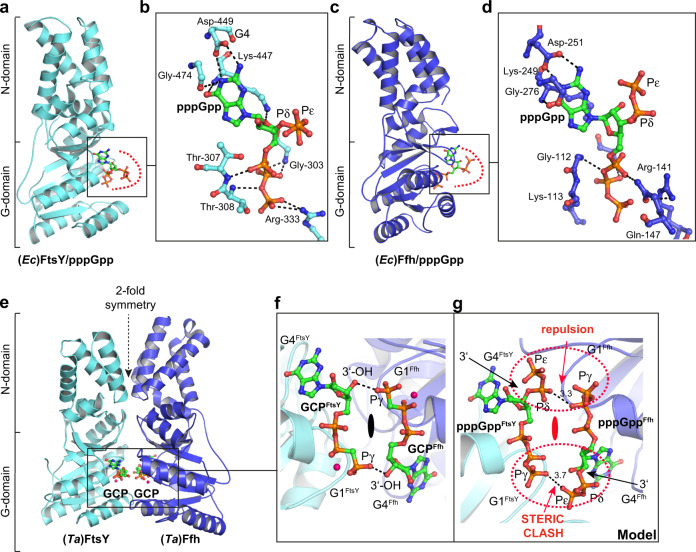
(p)ppGpp binds in the nucleotide-binding pocket of the Ffh and FtsY-NG domains. **a** Overall topology of (*Ec*)FtsY-NG in complex with pppGpp. **b** Zoom into the active site of (*Ec*)FtsY-NG bound to pppGpp highlighting the residues involved in ligand binding. **c** Overall topology of (*Ec*)Ffh-NG in complex with pppGpp. **d** Detailed view of the active site and the residues involved in binding of pppGpp. **e** Overall topology of the Ffh/FtsY-NG domain heterodimer from Thermus aquaticus (Ta) bound to two GCP (GppCp, a non-hydrolysable GTP-analog) molecules mimicking the binding of GTP in the twinned nucleotide-binding site (PDB-ID: 1OKK)^32^. **f** Zoom into the nucleotide-binding pocket shared between the NG domains of Ffh and FtsY. The 3’-OH of one GCP molecule interacts with the γ-phosphate of the opposing GCP molecule and vice versa. **g** Overlay of the Ffh/FtsY heterodimer with the pppGpp-bound structures of (*Ec*)Ffh and (*Ec*)FtsY (this study). Close up of the shared nucleotide-binding site shows that the δ- and ε-phosphates at the 3′-OH position of the ribose moiety of (p)ppGpp will lead to charge repulsion, where the hydrogen bond is formed in the heterodimer. Reciprocal arrangement of the two GTP nucleotides in the shared catalytic side of the Ffh/FtsY heterodimer would thereby be prevented.

To gain a molecular understanding of the (p)ppGpp inhibition of the GTP-dependent SRP/FtsY-targeting complex formation, we decided to structurally analyze the (p)ppGpp-bound states of Ffh and FtsY. We determined the crystal structures of the pppGpp-bound NG domains of (*Ec*)Ffh and (*Ec*)FtsY at resolutions of 2.5 and 2.4 Å, respectively (Fig. 4a–d; Supplementary Fig. 5 and Supplementary Table 1), as well as the crystal structures of (*Bs*)Ffh and (*Ec*)Ffh in complex with ppGpp and Mg^2+^ at resolutions of 2.5 and 2.8 Å, respectively (Supplementary Table 1; Supplementary Figs. 5 and 6). Each of the alarmones could be unambiguously identified by and placed into the available densities (Supplementary Fig. 5). The ppGpp or pppGpp molecules are bound to the G domains of Ffh and FtsY and occupy the canonical guanosine nucleotide-binding site (Fig. 4a–d and Supplementary Fig. 6). In detail, the guanine moiety of (p)ppGpp is recognized by the well-described aspartate of the G4 element responsible for the discrimination of guanosine nucleotides by P-loop type GTPases (Fig. 4b, d and Supplementary Fig. 6b, d). The α-, β-, and γ-phosphates of (p)ppGpp are coordinated by the G1-element (P-loop), and by residues originating from the switch I and II regions including the G2 and G3 elements, respectively. The δ- and ε-phosphate, which are covalently bound to the 3′-OH group of the ribose moiety and discriminate (p)ppGpp from its GDP/GTP counterparts, point away from the active sites of both GTPases (Fig. 4b, d). Both phosphates are not coordinated or form any obvious contacts to the GTPases of Ffh and FtsY (Fig. 4b, d). The missing coordination leads to a high flexibility of the δ- and ε-phosphate, as observed in the crystals of (*Ec*)Ffh with pppGpp (Supplementary Fig. 5e, f). We conclude that ppGpp and pppGpp interact with the SRP-GTPases Ffh and FtsY in the same way as its counterparts GDP and GTP, respectively, also providing the structural explanation for their comparable *K*_D_ values (see above; Fig. 3e).

Previous structural analysis showed that the essential targeting complex forms through a strictly GTP-dependent, pseudo-symmetric heterodimer of the NG domains of Ffh and FtsY (Fig. 4e)^31,32,50,51^. In this complex, the G domains of Ffh and FtsY form a shared catalytic center into which each GTPase provides one GTP molecule (Fig. 4f). These two GTP molecules reciprocally interact with each other in a way that the 3′-OH group of the ribose moiety of one GTP interacts with the γ-phosphate moiety of the other, and vice versa (Fig. 4f). The tight and reciprocal arrangement of both GTPs via their 3′-OH groups is essential for complex association, reciprocal GTPase activation and catalysis^31^. Our structural analysis of (p)ppGpp-bound Ffh or FtsY now shows that this reciprocal nucleotide arrangement is no longer possible, when the δ-, ε-pyrophosphate moieties at the 3′-OH group of the alarmones prevent the formation of the crucial hydrogen bond, and by introducing electrostatic repulsion by the negatively charged phosphates (Fig. 4g). This structural view explains our biochemical observation that (p)ppGpp efficiently hinders formation of the Ffh/FtsY complex, and all subsequently associated steps such as the reciprocal stimulation of Ffh and FtsY GTPase activity during interaction with the SecYEG complex.

### pppGpp affects the conformational flexibility of SRP-bound to RNCs in the Gram-positive model organism *B. subtilis*

**Fig. 5:**
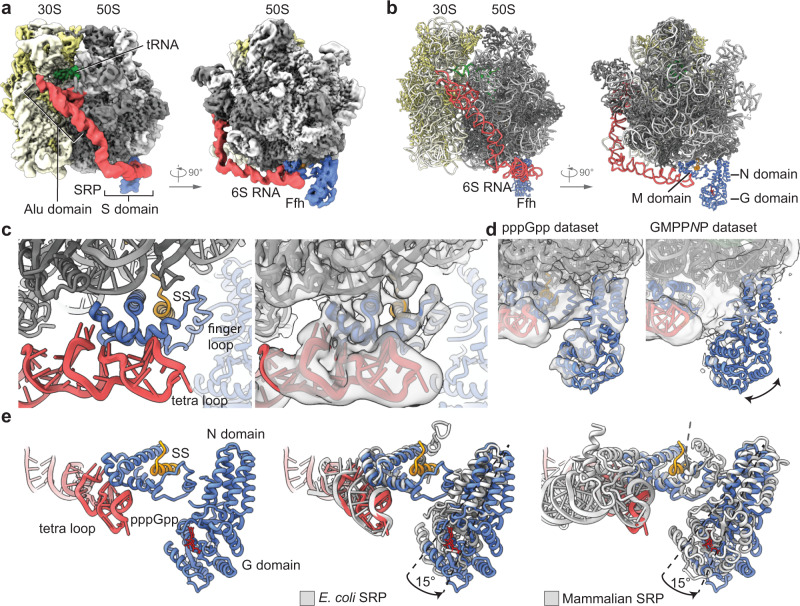
Cryo-EM structures of SRP-RNC complexes. **a** pppGpp dataset cryo-EM map of (*Bs*)SRP-bound MifM-stalled RNCs filtered at local resolution, small 30S subunit in yellow and large 50S subunit in gray, SRP 6S RNA in red and Ffh in blue. SRP consists of two functional domains: the signal sequence recognition domain (S domain) and the translational elongation arrest domain (Alu domain). The S domain of the SRP is homologous to the domains II–IV of the eukaryotic 7S RNA and carries the Ffh subunit whereas the Alu domain resembles the domain I of the 7S RNA and binds to the GTPase center of the ribosome^21^. **b** Molecular model of (*Bs*)SRP-bound MifM-stalled RNCs. **c** Zoomed view of the Ffh-M domain with signal sequence (SS) bound and transparent cryo-EM density in gray (right). **d** Comparison of Ffh-NG domain cryo-EM density for the pppGpp and GMPP*N*P dataset. **e** Zoomed view of the S domain of SRP and position of nucleotide-binding site in the Ffh-NG domain (left); comparison with previously found positions of the NG domain in bacteria (PDB 5GAF, *E. coli*) and eukaryotes (PDB 3JAJ, *Oryctolagus cuniculus*) (right). Structures are aligned on the large ribosomal subunit.

So far, we could show that (p)ppGpp hinders SRP-mediated protein targeting at the level of the GTP-dependent SRP-FtsY-targeting complex formation. However, this does not exclude that pppGpp may already affect the RNC-bound SRP through its binding to the Ffh-NG domain. The (*Bs*)Ffh-NG domain also bound ppGpp and pppGpp with similar affinities as their GDP and GTP counterparts, respectively (Fig. 3e; Supplementary Figs. 3 and 4). Next, we analyzed SRP bound to (*Bs*)MifM-stalled RNCs, and (*Ec*)TnaC-stalled RNCs bearing the FtsQ transmembrane segment (TM) in the NC in the presence of either pppGpp or GMPP*N*P by cryo-EM. Using a *B. subtilis* cell-free system we translated the MifM-stalling mRNA, which contains the MifM leader peptide with shortened C-terminus, a defined ribosome stalling site, the MifM N-terminal TM and a cleavable His-tag^52^. Stalled RNCs were isolated via affinity purification and sucrose density gradient centrifugation. Subsequently, the RNCs were reconstituted with recombinant (*Bs*)SRP (Ffh and 6S RNA), in the presence of either pppGpp or GMPP*N*P. Cryo-EM analysis revealed stalled 70S ribosomes with a P-site tRNA and a nascent chain in the peptide exit tunnel as observed before^52^. The GMPP*N*P and pppGpp datasets contained stably bound SRP in 24% and 38% of the particles, respectively (Supplementary Fig. 7 and Supplementary Table 2), which overall resembled the previously observed RNC-SRP complex^53^. However, in the previously observed (*Bs*)RNC-SRP reconstruction in the absence of nucleotides^53^, but also in the new RNC-SRP-GMPP*N*P reconstructions (*this study*, Supplementary Fig. 7), the Ffh-NG domain of SRP was largely delocalized. In contrast, the pppGpp dataset revealed the Ffh-NG domain in a stable conformation for the majority (57%) of the SRP-containing particles (Supplementary Fig. 7). This final class was then refined to an average resolution of 3.3 Å with local resolution ranging from below 3.0 Å in the ribosomal core to 8-10 Å for SRP and the periphery of the ribosome (Fig. 5a, b and Supplementary Fig. 8a, b). Structural analysis of the SRP M domain showed an open conformation (Fig. 5c) with a rod-like density representing the signal sequence bound in a position very similar to previously observed structures^53–57^. The M domain contacted the 23S rRNA H24 via helix3 (Arg-399) and helix4 (Asn-414, Gln-411) and is additionally positioned by multiple interactions with the 6S RNA, which in turn interacts with H100 of the 23S rRNA (Fig. 5c).

In the GMPP*N*P dataset, a final SRP-containing class was refined to an average resolution of 3.0 Å (Supplementary Fig. 8c, d). Here, no distinct conformational sub-states could be resolved for the Ffh-NG domain and all attempts to sub-classify the data with a mask around the S domain did not reveal any distinct NG domain density (Supplementary Fig. 7). These observations were similar as before for the (*Bs*)RNC-SRP apo complex in the absence of any nucleotides^53^. In both structures, the apo and the GMPP*N*P-bound one, flexibility of the NG domain coincided with a higher degree of flexibility also of the Ffh-M domain, which in contrast to the pppGpp structure lacked clear density for the signal sequence (SS), the flexible finger loop and the GM linker connecting M and NG domains. Taken together, the presence of pppGpp results in a stabilization of the NG domain conformation of Ffh on the *B. subtilis* RNC after signal sequence recognition, which might already provide a steric problem for the initial phase of heterodimer formation (Fig. 5d).

### pppGpp seems not to affect the conformational flexibility of SRP-bound to RNCs in the Gram-negative model organism *E. coli*

In contrast to the Gram-positive bacterium *B. subtilis*, the Gram-negative model bacterium *E. coli* contains an SRP with an approximately 200 bases shorter SRP-RNA, while the Ffh protein is highly conserved between both model organisms. Thus, we also investigated whether pppGpp would induce a similar stabilization of the Ffh-NG domain with *E. coli* RNCs as we observed for the *B. subtilis* system (see previous chapter). *E. coli* RNCs were prepared by in vivo translation using a construct containing a N-terminal cleavable His-tag, the FtsQ-TM and the TnaC-stalling sequence^58,59^. As for the *B. subtilis* samples, the RNCs were isolated via affinity purification, sucrose density gradient centrifugation, and reconstituted with recombinant (*Ec*)SRP (Ffh and 4.5S RNA) in the presence of either pppGpp or GMPP*N*P. Cryo-EM analysis revealed stalled 70S ribosomes with a P-site tRNA and a nascent chain in the peptide exit tunnel. Both datasets were classified for the presence of SRP and NG domain. However, no difference in the conformation of the NG domain between the pppGpp and GMPP*N*P treated samples could be observed (Supplementary Fig. 9). As a second approach to facilitate an unbiased comparison, the GMPP*N*P and pppGpp datasets were combined and sorted for the presence of SRP together (Supplementary Fig. 10). After this joined classification, SRP-containing particles (Supplementary Fig. 10 and Supplementary Table 3) were segregated again into the GMPP*N*P and pppGpp datasets, and refined to an average resolution of 3.2 and 3.1 Å, respectively. Local resolution for SRP ranges between 5 and 12 Å with the lowest resolution in the NG domain region indicating flexibility (Supplementary Fig. 11). Also, by using this strategy and in contrast to the *B. subtilis* system, no significant difference in the overall SRP binding as well as in the somewhat flexible NG domain position could be observed. Overall, both structures and the position of the NG domain were in agreement with previously observed *E. coli* SRP-bound ribosome cryo-EM structures^54^. Thus, it appears that pppGpp does not affect the conformation of the SRP bound to RNCs in *E. coli*.

In *B. subtilis*, the presence of pppGpp results in stabilization of the Ffh-NG domain conformation on the RNC after signal sequence recognition, which might already provide a steric problem for the initial phase of heterodimer formation (Fig. 5d). In contrast to (*Bs*)SRP, the NG domains of Ffh or SRP54 in RNC-bound *E. coli* or mammalian SRP, respectively, display a less flexible binding to the ribosome independent of nucleotides. While the (*Bs*)SRP N domain contacted the ribosomal protein uL29 through NG loops 1 and 2 similar to *E. coli* and mammalian SRP^54–57^, the position of the G domain has changed by ~13 Å as a result of a rotation of the entire NG domain by about 15 degrees (Fig. 5e). Yet, comparison of the position of (*Bs*)SRP with these structures showed a different binding mode, in case of the pppGpp-bound state (*Bs*)SRP rotated away from the 6S RNA tetra-loop and the Ffh-M domain (Fig. 5e).

## Discussion

**Fig. 6:**
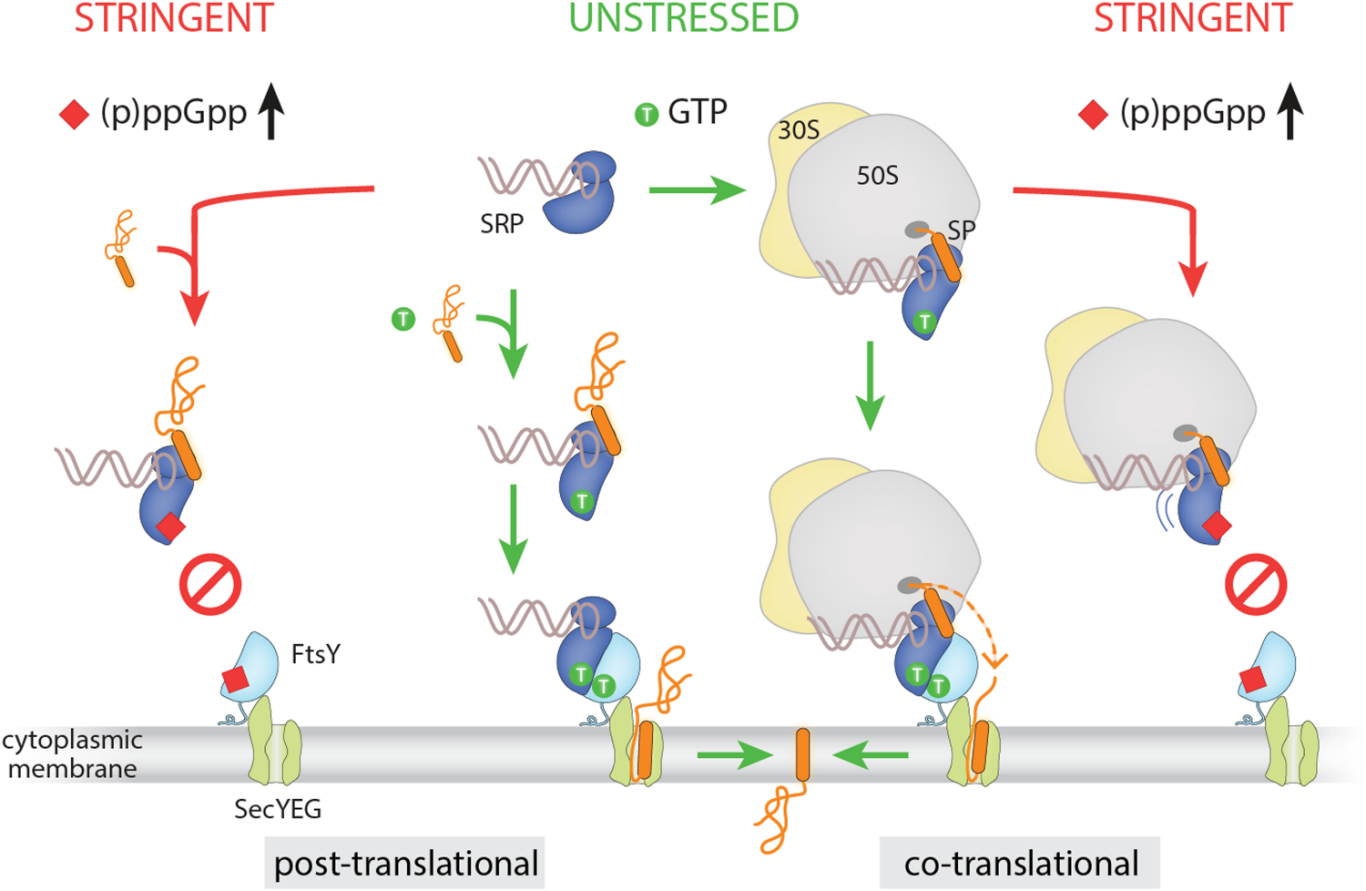
Schematic summary of the bimodal interference of the alarmones (p)ppGpp with the post- and co-translational SRP-dependent membrane-targeting pathway. In unstressed cells, SRP (Ffh in blue and SRP RNA in gray) usually recognizes a signal peptide (SP, orange) at the ribosomal exit tunnel (co-translational) but can recognize some proteins also after they have been released from the ribosome (post-translational). Binding of a GTP (green) to both SRP and the SRP receptor FtsY (light blue) then allows the formation of the SRP-FtsY-targeting complex, which leads to stimulation of GTP hydrolysis and transfer of the RNC to the SecYEG translocon (light green). In contrast, under stringent stress conditions, (p)ppGpp (red) binds to SRP and prevents formation of the SRP-FtsY-targeting complex through steric hindrance both during post- and co-translation membrane targeting.

Adaptation to stress conditions requires adjustable regulatory networks and signaling mechanisms that enable bacterial cells to survive threatening nutrient limitations and other environmental extremes. In this study, we shed light on an additional regulatory role of the stress signaling alarmones (p)ppGpp. We link the bacterial stress and starvation triggered (p)ppGpp response to the negative regulation of the SRP machinery required for the insertion into and the secretion of proteins across the cytoplasmic membrane. Our study unravels the molecular mechanism by which the alarmones (p)ppGpp can restrict the insertion of membrane proteins through the SRP-mediated co- and post-translational-targeting pathways (Fig. 6).

### Mechanism of (p)ppGpp-dependent inhibition of the SRP-system

The alarmones ppGpp and pppGpp bind to the GTPases of Ffh and FtsY with binding affinities in the low μM range, closely reflecting the affinities of their counterparts GDP and GTP, respectively (Fig. 3e; Supplementary Figs. 3 and 4). Consequently, both alarmones can act as competitive inhibitors of GDP and especially GTP, which is critically required to enable interaction of SRP with its receptor. Both interact via their NG domains, which form a composite active site in which two GTP molecules reciprocally align, such that the 3′-OH group of one GTP interacts with the γ-phosphate of the other, and vice versa^31–33^. However, when either Ffh or FtsY or both are bound to (p)ppGpp, this reciprocal arrangement of the two GTPs within the Ffh-FtsY heterodimer is no longer possible. The δ-, ε-pyrophosphate moieties at the 3′-OH groups of ppGpp and pppGpp prevent the formation of this crucial nucleotide arrangement^31^, and additionally introduce an electrostatic repulsion through the negatively charged phosphates (Fig. 4g). Consequently, SRP-receptor formation and the subsequent stimulation of both GTPases are impaired, as shown in this study.

We also investigated whether the pppGpp would already impact SRP at a RNC presenting a signal sequence, prior to the interaction of SRP with the receptor. We analyzed the SRP particles of *B. subtilis* and *E. coli*, both of which strongly differ in the length of the SRP RNA, in the context of their cognate RNCs. To our surprise, we found that in *B. subtilis*—but not in *E. coli*—SRP is stabilized by pppGpp on the RNC in an unusual and rather rigid conformation with the NG domain more distant from the SRP RNA compared to other RNC-SRP complexes. This restricted mobility suggests that in *B. subtilis* pppGpp may already inhibit the earliest step in the targeting process that immediately follows recognition of the signal sequence. During this step, facilitated by the conserved tetra-loop of the SRP RNA, FtsY would usually engage in the first SRP-NG domain interaction to initiate productive heterodimer formation. The restricted mobility of the pppGpp-bound NG domain of SRP could possibly prevent this productive early interaction with FtsY, thereby potentially adding a second layer of inhibition of the secretory pathway. However, these observations require further investigation. Moreover, we did not observe a similar pppGpp-dependent stabilization of the RNC-bound SRP in the *E. coli* model system. While we cannot explain the structural differences between *B. subtilis* and *E. coli* due to limited local resolution of our reconstructions, this potential extra layer of inhibition might not exist in *E. coli*. Why that is so requires further clarification.

### Physiological considerations

Intracellular (p)ppGpp concentrations can raise from low basal levels (appr. 10–40 μM) during logarithmic growth^43,44^ up to 800 μM when cells enter stationary phase^60^. Moreover, in circumstances of acute amino acid starvation intracellular alarmone levels peak at appr. 1 mM^43,47,61,62^. Hence, different targets (with varying affinities) are regulated over a gradient of (p)ppGpp concentrations during the growth of a bacterial population, while very high concentrations of (p)ppGpp (appr. 1 mM) result in growth arrest^63–65^. A detailed view on (p)ppGpp targets shows that the binding affinities vary between low μM range, e.g., for RNA polymerase, and many ribosome biogenesis factors (Era, ObgE, or RbgA) up to a few hundred μM in the case of the DNA primase DnaG or proteins involved in carbon metabolism (overview in ref. ^15^). Such a gradual system allows the cells to fine tune metabolic processes and balance fluxes in response to changes in nutrient availability and other stressful conditions. Importantly for targets that are bound competitively by (p)ppGpp and GTP, the intracellular concentration of GTP impairs the fraction of (p)ppGpp-bound proteins. While the intracellular GTP concentration in *E. coli* during normal, unstressed growth conditions varies between 1-5 mM, it is highly reduced during stringent conditions caused by the inhibition of GTP anabolism through increasing (p)ppGpp concentrations^43–45^. This negative correlation has also been described in *B. subtilis*^46–48^. Consequently, inhibition of GTPases is also dependent on the GTP to (p)ppGpp ratio. This relation is e.g., reflected in the inhibition of the GTPase RbgA involved in ribosome biogenesis, where an inhibitor constant K_i_ of 300 μM (ppGpp) and 500 μM (pppGpp) has been determined^66^. The major (p)ppGpp synthetases Rel (in *B. subtilis*) and RelA (in *E. coli*) require the binding of deacetylated amino acids and N-terminal association to the ribosome to enable full activation of (p)ppGpp production^9,12,67^. Hence, (p)ppGpp regulation may not only be dependent on global pools, but also on local pools present in proximity of the ribosome-Rel complex. This local production of (p)ppGpp will inevitably influence the GTPases of the SRP/FtsY-targeting machinery. During harsh environmental conditions bacterial cells may use the shutdown of essential pathways such as transcription (RNA pol)^68^, ribosome biogenesis (Era, ObgE, or RbgA)^66,69–71^, translation^60,72^ and also SRP-dependent membrane targeting through the stringent response alarmones as a pausing mechanism. It allows microorganisms to slow down their cellular processes and metabolisms, rather entering a persistence-like state to preserve the ability to recover when conditions are favorable again. Hence, inhibition of the SRP pathway might be an additional layer of cellular control and adaptive pausing to survive during stressful times.

## Methods

### In vitro synthesis of 4.5S and 6S RNA

For in vitro synthesis of 4.5S RNA, pT7/T3α19, carrying the 4.5S RNA coding sequence^73^ was linearized using BamHI. In vitro transcription was performed using the AmpliScribe T7-Flash Transcription kit (Epicentre Biotechnologies, Madison, USA). The 4.5S RNA was purified using the RNA purification Kit (Qiagen, Hilden Germany) and stored at −80 °C. For in vitro synthesis of 6S RNA, the plasmid pUC19 coding for (*Bs*) 6S RNA^53^ was linearized using restriction enzyme HindIII HF (NEB). Two micrograms of DNA and in house-prepared T7 polymerase were incubated in 5 mM DTT, 8 mM ATP, CTP, GTP, UTP, 10x T7 buffer (400 mM Tris pH 7.9, 25 mM spermidine 260 mM MgCl_2_, 0.1% (v/v) Triton X-100) at 37 °C for 3 h. After in vitro transcription, the RNA was precipitated using phenol/chloroform, resuspended in water, and stored at −80 °C.

### Plasmid construction and protein purification

Protein purification of full-length constructs originating from *E. coli* followed previously described protocols for SecYEG^74^, full-length (*Ec*)Ffh^74^ and (*Ec*)FtsY^75^. In brief, for (*Ec*)Ffh and (*Ec*)FtsY purification, expression was induced by adding 1 mM IPTG and cells were broken using a French Pressure cell (Thermo Fisher Scientific, Schwerte, Germany). Proteins were purified via their His-tags using an Äkta chromatography system using a HisTrap FF nickel column (GE Healthcare, Waukesha, WI, USA). SecYEG was purified after arabinose induction (0.5%) from *E. coli* cells carrying pBAD-SecYE_His_G. Membranes were isolated, solubilized with 1% dodecyl maltoside and purified via Talon resin (Takara, St. Germaine-en-Laye, France). For overexpression and purification of the (*Ec*)Ffh- and (*Ec*)FtsY-NG domains, the coding gene fragments from *E. coli* were amplified by polymerase chain reaction (PCR) and cloned into pET24d (Novagen) via the NcoI/XhoI (FtsY) or NcoI/HindIII (Ffh) restriction sites (primers are listed in Supplementary Table 4). This resulted in the plasmids pNM103 and pNM101, respectively. Both proteins contained a C-terminal hexa-histidine (His_6_) tag. The gene fragment encoding the Ffh-NG domain from *B. subtilis* was amplified by polymerase chain reaction (PCR) and cloned into a pET24d (Novagen) vector modified for modular cloning via BsaI restriction sites (primers are listed in Supplementary Table 4). This resulted in the plasmids pLC163. (*Bs*)Ffh-NG also contained a N-terminal hexa-histidine (His_6_) tag. Proteins derived from *E. coli* were produced in *E. coli* BL21 (DE3) (Novagen). Four liters of LB medium containing (50 µg mL^−1^ kanamycin) and 1% (w/v) lactose for autoinduction of the P_*lac*_ promoter driving the T7 polymerase required for recombinant gene expression were incubated in an aerial shaker for 18 h at 30 °C overnight. Proteins derived from *B. subtilis* were produced in *E. coli* Rosetta pLysS (Novagen). 4 liters of LB medium containing (50 µg mL^−1^ kanamycin) were inoculated with an overnight culture to an OD_578_ of 0.08 and incubated at 37 °C in an aerial shaker (180 rpm). When cultures reached OD_578_ of 0.5 overproduction of the recombinant proteins was induced by the addition of 1 mM IPTG and the cultures were further incubated for 3 h. After harvesting, cells were lysed by a Microfluidizer (M110-L, Microfluidics). The lysis buffer contained 20 mM HEPES-Na (pH 8.0), 250 mM NaCl, 20 mM KCl, and 50 mM imidazole. Cell debris was then removed by high-speed centrifugation for 20 min at 48,000 × *g*. All proteins were initially purified by Ni-ion affinity chromatography, eluting in lysis buffer containing 250 mM imidazole. 30 mM (final concentration) ethylenediaminetetraacetic acid (EDTA) was subsequently added and incubated for 10 min at room temperature. Anion-exchange chromatography (MonoQ 5/50 GL, GE Healthcare) was conducted utilizing a linear gradient of buffer A (20 mM HEPES-Na (pH 7.5), 20 mM EDTA, 100 mM NaCl and buffer B (buffer A containing 1 M NaCl) over 20 column volumes (CV). The eluted proteins were concentrated (10 kDa MWCO) and further polished by size-exclusion chromatography on a S200 XK26 column or a S200 XK16 column (GE Healthcare) with SEC buffer consisting of 20 mM HEPES-Na (pH 7.5), 200 mM NaCl, 20 mM KCl, and 20 mM MgCl_2_. Purified proteins were analyzed for the presence of remaining bound nucleotides using a standard nucleotide HPLC method as described below.

The plasmid pET46 coding for full-length (*Bs*)Ffh with N-terminal 6xHis-tag and HRV 3C cleavage site was transformed in *E. coli* strain BL21(DE3). Cells were grown in LB medium to mid-log phase (OD_600_ = 0.6) at 37 °C and induced with 1 mM IPTG at 16 °C for 20 h. Cells were harvested by centrifugation at 5471 × *g* and 4 °C for 8 min, washed with buffer A (25 mM HEPES/KOH pH 7.5, 500 mM KCl, 10 mM imidazole, 1 mM DTT, 0.1 mM EDTA, 0.1 mM PMSF, 1:1000 protease inhibitor (pill mL^−1^), 10% (v/v) glycerol) and frozen in liquid nitrogen. Frozen cells were ground using a Spex SamplePrep Freezer Mill and the powder stored at −80 °C until further use. After 15 g of cell powder was thawed in 100 mL buffer A, cell debris was removed by centrifugation at 30,597 × *g* for 20 min. The cleared lysate was then incubated with 6 mL of prewashed TALON metal affinity resin for 2 h. Afterwards the beads were washed with 15 (CVs buffer A, 7 CVs buffer B (25 mM HEPES/KOH pH 7.5, 1,000 mM KCl, 10 mM imidazole, 1 mM DTT, 0.1 mM PMSF, 1:1000 protease inhibitor (pill mL^−1^), 10% (v/v) glycerol) and 3 CVs buffer A without protease inhibitor. The beads were then incubated with 0.22 mg mL^–1^ 3 C protease in buffer C (25 mM HEPES/KOH pH 7.5, 500 mM KCl, 1 mM DTT, 10% (v/v) glycerol) overnight on a wheel. The elution was diluted 1:10 in buffer D (25 mM HEPES/KOH pH 7.5, 100 mM KCl, 1 mM DTT, 10% (v/v) glycerol) and loaded with 1 mL min^–1^ flow rate on a HiTrap SP HP cation-exchange chromatography column. The column was washed with 5 CVs buffer D and elute over a 4 CVs gradient from 0-100% buffer E (buffer D with 1000 mM KCl). 1 mL fractions were collected and analyzed by sodium dodecyl sulfate–polyacrylamide gel electrophoresis (SDS-PAGE). Ffh-containing fractions were pooled and concentrated to 1 mL using an Amicon 30k MWCO and subjected to size-exclusion chromatography using a Superdex 200. Again, Ffh-containing fractions were pooled, concentrated, and used for reconstitution of SRP.

### In vitro protein synthesis, protein purification, and generation of proteoliposomes

For protein transport assay, YohP cloned in pET19b was synthesized in vitro using a purified transcription/translation system composed of cytosolic translation factors (CTF) and high salt-washed ribosomes^35,36^. The ^35^S-Methionine/^35^S-Cysteine labeling mix was obtained from Perkin Elmer (Wiesbaden, Germany). INVs of *E. coli* cells were prepared by sucrose gradient centrifugation of cell extracts as described^76^ and resuspended in INV buffer (50 mM triethanolamine acetate, pH 7.5, 200 mM sucrose, 1 mM DTT). After in vitro synthesis, samples were incubated for 10 min at 37 °C with 35 µg mL^−1^ chloramphenicol for inhibiting translation and then centrifuged for 30 min at 186,000 × *g* in a Beckmann TLA55 rotor for removing ribosomes. The supernatant containing YohP was then incubated with INV, liposomes or SecYEG-proteoliposomes for 10 min at 37 °C in the presence of 10 µM GTP. When indicated, (p)ppGpp dissolved in 50 mM triethanolamine acetate, pH 7.5 was added during the incubation step. After incubation, one half of the reaction was directly precipitated with 10% (w/v) trichloroacetic acid (TCA), while the other half was first treated with 0.5 mg mL^−1^ proteinase K for 15 min at 25 °C and only then precipitated with TCA. Proteinase K was inactivated in 10% (w/v) TCA by incubation for 10 min at 56 °C. Next, the samples were denatured at 56 °C for 10 min in 35 μL of TCA loading dye (prepared by mixing one part of solution III (1 M dithiothreitol) with 4 parts of solution II (6.33% (w/v) SDS, 0.083 M Tris, 30% (v/v) glycerol and 0.053% (w/v) bromophenol blue) and 5 parts of solution I (0.2 M Tris, 0.02 M EDTA pH 8)) and analyzed after separation on a modified SDS-PAGE^35^ by phosphor imaging. For quantification of YohP insertion, autoradiography samples were analyzed by using the *ImageQuant* (GE Healthcare) software. All experiments were performed three times as independent biological replicates and representative images are shown. Mean values and SEM values were determined by using either Excel (Microsoft Corp.) or GraphPad Prism (GraphPad Prism Corp. San Diego). (*Ec*)Ffh (full-length) was concentrated on a 10 kDa centrifugal filter (Amicon Ultra, Witten, Germany) and re-buffered in HT buffer containing 50% (v/v) glycerol (50 mM HEPES, pH 7.6, 100 mM KOAc, pH 7.5, 10 mM Mg(OAc)_2_, 1 mM DTT) using a PD-10 column (GE Healthcare, Munich Germany). The protein was stored at −20 °C. (*Ec*)FtsY (full-length) was re-buffered in HT buffer using a PD-10 Column (GE Healthcare, Munich, Germany) and stored at −80 °C. SRP was reconstituted by incubating 1.5 µM (*Ec*)Ffh with 0.1 mg mL^−1^ 4.5S RNA (see above) for 15 min at 25 °C in HT buffer. *E. coli* phospholipids were purchased from Avanti polar lipids, Inc (Alabaster, USA) and liposomes were generated as described^77^, representing a phospholipid composition of 70% phosphatidylethanolamine (PE), 25% phosphatidylglycerol (PG) and 5% cardiolipin (CL). SecYEG-proteoliposomes were created as described^74,78^. In brief, 200 µg of liposomes and 14-16 µg of purified (*Ec*)SecYEG were prepared in 150 µL buffer (50 mM triethanolamine acetate (TeaOAc), pH 7.5, 1 mM DTT, and 1.5% octyl-glycoside. The samples were dialyzed with PL-buffer (50 mM TeaOAc, pH 7.5, 1 mM DTT), pelleted and resuspended in PL-buffer to a final protein concentration of 100 ng µL^−1^ and stored at −80 °C. Before each use, proteoliposomes were briefly sonicated. In in vitro protein transport assays, 1 µL liposomes or proteoliposomes were used per in vitro reaction.

### Floatation analyses of FtsQ and LepB ribosome-associated nascent chains (RNCs)

For in vitro synthesizing FtsQ- and LepB-RNCs, the T7-dependent expression vectors pKSM-FtsQ and pKSM-LepB were used^37,79^. In brief, the first 102 amino acids of LepB, and the first 120 amino acids of FtsQ were fused to the SecM stalling sequence and cloned into the pET19b vector. In vitro synthesis was performed in a coupled transcription/translation system as described for YohP. After in vitro synthesis, the RNCs were incubated with INVs (1 µL), liposomes (2 µL), or SecYEG-proteoliposomes (2 µL; 100 ng SecYEG µL^−1^) in floatation buffer (50 mM triethanolamine acetate, pH 8.0; 10 µM GTP, 10 mM magnesium acetate, 70 mM potassium acetate; 250 mM sucrose and 1 mM DTT) for 15 min at 25 °C. When indicated, SRP/FtsY (20 ng µL^−1^ each) and ppGpp or pppGpp (50 µM final concentration) was present during incubation. Membrane binding of RNCs was assayed by floatation analyses^39^. The reaction mixture was adjusted to 1.6 M sucrose (final volume 100 µL) and overlaid with 200 µL of 1.25 M sucrose and 100 µL 0.25 M sucrose, each prepared in floatation buffer. Following centrifugation in a TLA 100.2 rotor (Beckmann-Coulter) at 43,4902 × *g* for 90 min, the upper 200 µL of the gradient, containing the membrane fraction (MF) were withdrawn and precipitated with 10% (w/v) TCA (final concentration). The pelleted (non-bound soluble fraction, SF) RNCs were resuspended in the remaining 200 µL of the gradient and TCA precipitated. Samples were subsequently analyzed by 15% SDS-PAGE and autoradiography. RNC binding to membranes was quantified by using the *ImageQuant* (GE Healthcare, München, Germany) software. The experiments were performed at least three times as independent experiments and representative images are shown. Mean values and SEM values were determined by using either Excel (Microsoft Corp.) or GraphPad Prism (GraphPad Prism Corp. San Diego).

### Analytical size-exclusion chromatography (SEC)

For the analytical SEC, purified NG domains of (*Ec*)FtsY and (*Ec*)Ffh were diluted in a buffer containing 20 mM HEPES (pH 7.5), 200 mM NaCl, 20 mM MgCl_2_ and 20 mM KCl to a final concentration of 100 µM (Fig. 3g) and 50 µM (Fig. 3h, i) each. Indicated amounts of nucleotides were added simultaneously, and the solution was incubated for 30 min at room temperature. 100 µL were then injected at 4 °C on to a pre-equilibrated S200 300/10 GL analytical size-exclusion column (GE Healthcare, München, Germany) on an Äkta system (UNICORN 7.6; Cytiva). Data has been plotted using GraphPad Prism (GraphPad Prism Corp. San Diego).

### Isothermal titration calorimetry (ITC)

Ligands and proteins (purified NG domains of (*Ec*)Ffh, (*Ec*)FtsY and (*Bs*)Ffh) were diluted with a buffer containing 20 mM HEPES-Na (pH 7.5), 200 mM NaCl, 20 mM MgCl_2_ and 20 mM KCl. The NG domains of (*Ec*)Ffh, (*Ec*)FtsY and (*Bs*)Ffh were titrated in the sample-cell at a nominal concentration of 25 µM each. The nucleotides GDP, GTP, ppGpp and pppGpp (Jena Bioscience, Germany) were placed in the syringe and their concentrations were predetermined by absorbance at 252 nm to saturate the protein samples during the titrations. All the measurements were performed at 25 °C with the instrument MicroCal PEAQ-ITC (©Malvern Panalytical) with a method consisting of 13 injections (first 0.4 µL, and the rest 3 µL each) and 150 s of spacing. The raw data (see source) were processed with the MicroCal PEAQ-ITC Analysis Software (©Malvern Panalytical) using the “one set of sites” models.

### Microscale thermophoresis

Ligand binding assays with purified NG domains of (*Ec*)Ffh, (*Ec*)FtsY and (*Bs*)Ffh proteins were carried out by microscale thermophoresis (MST)^80^. All experiments were performed on a Monolith NT.115 (NanoTemper Technologies GmbH, Munich, Germany; software: NanoTemper Control Version 2.0.2.29) at 21 °C (red LED power was set to 50–100% and infrared laser power to 75%). After labeling of primary amines within (*Ec*)Ffh, (*Ec*)FtsY, and (*Bs*)Ffh (50 µM each) with the dye NT 647 (according to the manufacturers protocol), the proteins were re-buffered in SEC buffer containing 20 mM HEPES-Na, 20 mM KCl, 20 mM MgCl_2_, 200 mM NaCl (final pH 7.5), 10 mg ml^−1^ BSA and 0.007% Tween. Two-hundred nanomolar of (*Ec*)Ffh, (*Ec*)FtsY, and (*Bs*)Ffh were titrated with GDP, GTP, ppGpp, and pppGpp starting from a concentration ranging between 0.75 and 3 mM, respectively. At least six independent MST experiments per ligand and protein were recorded at 680 nm and analyzed using NanoTemper Analysis version 1.5.37 and 1.2.009, and Origin8G software suits.

### Determination of GTPase activity

GTPase activity of full-length (*Ec*)Ffh and the NG domain of (*Ec*)FtsY was assayed in a buffer containing 25 mM HEPES-K pH 7.5, 10 mM Mg(OAc)_2_, 300 mM K(OAc), 1 mM DTT, and 2.5% (v/v) glycerol. The samples contained 5 µM (*Ec*)Ffh, 5 µM (*Ec*)FtsY-NG, 6 µM 4.5S RNA, 5 µM ΔEspP (EspP signal peptide sequence: MKK HKR ILA LCF LGL LQS SYS WAK KKK, custom synthesized from Genosphere Biotechnologies (France)^40^, and 100 µM C_12_E_8_ (octaethylenglykol-monododecylether, Sigma Aldrich)^40^ as indicated in figures. Alarmones ppGpp (Jena Bioscience, ≥95% purity) or pppGpp (Jena Bioscience, ≥85% purity) were supplemented in concentrations of 10, 25, 100, 250, 1000, 2500, or 10,000 µM as indicated in the figures. The enzymatic reactions were initiated by the addition of 1 mM GTP (Jena Bioscience, ≥99% purity) and allowed to proceed for 60 min at 37 °C, after which 40 µL double-distilled water were added immediately followed by 150 µL of chloroform. The reaction tubes were then vigorously agitated for 5 s, heated up at 95 °C for 15 s and snap-frozen in liquid nitrogen. The tubes were thawn and, after centrifugation (17,300 × *g*, 10 min, 4 °C), an aliquot of the aqueous phase was withdrawn for analysis. The nucleotide content was determined by high-performance liquid chromatography (HPLC) on an Agilent 1260 Infinity system (software: ChemStation Rev. B04.03-SP1) equipped with a Metrosep A Supp 5–150/4.0 column (Metrohm). Ten microliters of sample were injected and nucleotides eluted isocratically at 0.6 mL min^−1^ flow rate of 100 mM (NH_4_)_2_CO_3_ pH 9.25 and detected at 260 nm wavelength. Commercial GDP and GTP solutions served as standards for the identification of the nucleotides based on their retention time. Data has been plotted using Microsoft Excel (version 14.6.8) and GraphPad Prism (GraphPad Prism Corp. San Diego).

### Crystallization and structure determination

Crystallization was performed by the sitting-drop method at 20 °C in 250-nL drops consisting of equal parts of protein and precipitation solutions. Protein solutions of 2.5–3 mM were incubated with 10 mM (final concentration) pppGpp or ppGpp, respectively, for 10 min at room temperature. Crystallization conditions were: (*Ec*)Ffh-NG with ppGpp and Mg^2+^ (0.2 M ammonium acetate, 20% (w/v) PEG 3350); (*Ec*)Ffh-NG with pppGpp (0.2 M sodium chloride, 0.1 M CHES pH 9.5, 50% (v/v) PEG 400); (*Ec*)FtsY-NG with pppGpp (8.5% (v/v) isopropanol, 0.085 M HEPES pH 7.5, 17% (w/v) PEG 4000, 15% (v/v) glycerol); (*Bs*)Ffh-NG with ppGpp and Mg^2+^ (0.1 M CHES pH 9.5, 30% (v/v) PEG 400). Prior to data collection, crystals were flash-frozen in liquid nitrogen using a cryo-solution that consisted of mother-liquor supplemented with 20% (v/v) glycerol. Data were collected under cryogenic conditions at the European Synchrotron Radiation Facility (Grenoble, France^81^) and at Deutsches Elektronen-Synchrotron (Hamburg, Germany). MxCube2 and MxCube3 were used for data collection (https://github.com/mxcube). Data were processed with XDS (version January 31, 2020) and scaled with XSCALE^82^. All structures were determined by molecular replacement with PHASER^83^, manually built in COOT^84^ (Coot Version 0.9.4.1), and refined with PHENIX^85^ (Phenix Version 1.17.1-3660 and 1.19). The search model for the Ffh structures was the *Thermus aquaticus* Ffh (PDB-ID: 3NG1^86^ https://www.rcsb.org/structure/3NG1). A structure of *E. coli* FtsY was already known and has been used as a model for molecular replacement (PDB-ID: 2YHS^27^ https://www.rcsb.org/structure/2YHS). Figures were prepared with Pymol (www.pymol.org)^87,88^.

### Reconstitution of (*Ec*)SRP and (*Bs*)SRP

For SRP reconstitution, the 6S SRP RNA was heated to 65 °C and then cooled down to 4 °C to allow proper folding. The 6S RNA and a tenfold molar excess of purified full-length (*Bs*)Ffh were then incubated together and loaded on a Superdex 200 using buffer F (25 mM HEPES/KOH pH 7.5, 300 mM KOAc, 10 mM Mg(OAc)_2_, 1 mM DTT, 2% glycerol). One milliliter fractions were collected and analyzed by SDS-PAGE and agarose gel. SRP-containing fractions were combined, concentrated and stored at −80 °C until further use.

### *B. subtilis* in vitro translation and reconstitution of SRP-bound RNCs

The MifM-encoding mRNA, which contains the MifM leader peptide with shortened C-terminus, a defined stalling site, the MifM N-terminal transmembrane segment (TM), a V5-tag and a cleavable His-tag, was prepared as described before by PCR amplification, DNA purification, in vitro transcription and phenol/chloroform precipitation^52^. The translation extract was prepared from the *B. subtilis* strain 168 ∆*hpf* ∆*ssrA* ∆*yjbM* ∆*ywaC*^89^. Cells were grown in LB medium supplemented with 1% (w/v) glucose, harvested at an OD_600_ between 0.6 and 0.8 and pelleted by centrifugation at 5471 × *g* and room temperature for 5 min. Afterwards, cells were resuspended in PBS (137 mM NaCl, 2.7 mM KCl, 10 mM Na_2_HPO_4_, 2 mM KH_4_PO_4_, pH 7.4), pelleted again by centrifugation at 5471 × *g* and 4 °C for 15 min and resuspended in lysis buffer (10 mM HEPES pH 8.2, 60 mM K-glutamate, 14 mM Mg(OAc)_2_). Cell lysis was performed using a microfluidizer (Microfluidics M-110L) and cell debris was removed by centrifugation at 30,597 × *g* and 4 °C for 20 min. The extract was aliquoted and frozen in liquid nitrogen. Activity of the extract as well as Mg buffer concentration was determined using the Luciferase Assay System (Promega).

The in vitro translation reaction was performed in 4 × 500 μL reaction volume. In all, 640 μL cell extract were mixed with energy buffer (final concentration in 2 mL: 2% (w/v) PEG 8000, 50 mM HEPES/KOH pH 8.2, 10 mM NH_4_OAc, 130 mM KOAc, 30 mM Na-pyruvate, 4 mM Na-oxalate, 50 µg mL^–1^ tRNA (from *E. coli*; Sigma 10109541 001), 0.2 mg mL^–1^ folinic acid, 0.1 µg mL^–1^ creatine kinase, 20 mM creatine phosphate, 4 mM ATP, 3 mM GTP, 0.1 mM amino acid mix, 1 mM DTT, 0.08 U SUPERase•In™ RNase Inhibitor (Invitrogen), 15 mM Mg(OAc)_2_). After heating the mixture to 32 °C for 2 min, 50 μg of mRNA were added to each aliquot and the in vitro translation was incubated at 32 °C for 40 min while shaking at 900 rpm. For affinity purification of ribosome-nascent chain complexes, the in vitro translation was incubated with 400 μL of prewashed TALON metal affinity resin for 45 min on a wheel. The flow-through was collected, beads were washed with 5 CVs buffer A (30 mM HEPES pH 7.5/KOH, 250 mM KOAc, 25 mM Mg(OAc)_2_, 20 mM imidazole, 0.1% DDM) and eluted by incubation for 2 h with 1 CV buffer B (30 mM HEPES pH 7.5/KOH, 250 mM KOAc, 25 mM Mg(OAc)_2_, 0.1% (w/v) DDM), 1.1 mg mL^–1^ 3 C protease). The sample was loaded on a 10–40% sucrose gradient (30 mM HEPES pH 7.5/KOH, 250 mM KOAc, 25 mM Mg(OAc)_2_, 0.1% (w/v) DDM, 10–40% (w/v) sucrose) and spun in a SW 40 Ti rotor (Beckman Coulter) at 54,322 × *g* for 16 h at 4 °C. The gradient was fractionated at a BioComp Gradient Station *ip* using a Triax Flow Cell for UV measurement. The 70S peak fractions were combined and RNCs were pelleted by centrifugation in a TLA110 rotor (Beckman Coulter) at 434,513 × *g* for 2 h at 4 °C. The pellet was resuspended in buffer C (25 mM HEPES pH 7.5/KOH, 150 mM KOAc, 10 mM Mg(OAc)_2_, 2 mM DTT), frozen in liquid nitrogen and stored at −80 °C. For reconstitution, RNCs and SRP were thawed on ice. SRP was incubated with either GMPPNP or pppGpp for 10 min at room temperature and with tenfold molar excess mixed with MifM-stalled RNCs. The final complex was incubated for 10 min at room temperature and subsequently analyzed by cryo-EM.

### *E. coli* translation in vivo and reconstitution of SRP-bound RNCs

The TnaC-stalled RNCs were prepared as previously described using the *E. coli* KC6 *∆ssrA ∆smpB* strain^58,59,90^. Cells were grown at 37 °C to an OD_600_ of 0.5 and expression of the of the RNC construct was induced with 0.2% arabinose. After 1 h, cells were harvested and resuspended in buffer A (50 mM HEPES pH 7.5/KOH, 250 mM KOAc, 25 mM Mg(OAc)_2_, 1 mM tryptophan, 0.1% (w/v) DDM, 250 μg ml^−1^ chloramphenicol and 0.1% EDTA-free complete proteinase inhibitors). Cells were lysed using a microfluidizer (Microfluidics M-110L) and centrifuged (5471 × *g*, 4 °C, 20 min). The cleared lysate was loaded on a sucrose cushion (buffer A + 750 mM sucrose) and spun in a Type 45 Ti Rotor (Beckman Coulter) for 20 h at 72,465 *g* and 4 °C. The pellet was resuspended in buffer B (50 mM HEPES pH 7.5/KOH, 250 mM KOAc, 25 mM Mg(OAc)_2_, 250 mM sucrose, 1 mM tryptophan, 0.1% (w/v) DDM, 250 μg ml^−1^ chloramphenicol and 0.1% EDTA-free complete proteinase inhibitors) and RNCs were isolated by incubation for 1 h with prewashed TALON metal affinity resin. The nascent chain consists of an N-terminal His-tag, an HRV 3 C protease cleavage site and the transmembrane segment of FtsQ (residues 4–51) followed by the stalling sequence of the tryptophanase leader peptide (TnaC). The amino acid sequence of the nascent chain is MGHHHHHHHH DYDIPTTLEV LFQGPGTAAL NTRNSEEEVS SRRNNGTRLA GILFLLTVCT TVLVSGWVVL GWMEDYPYDV PDYAGPNILH ISVTSKWFNI DNKIVDHRP*. The beads were washed with buffer C (50 mM HEPES pH 7.5/KOH, 500 mM KOAc, 25 mM Mg(OAc)_2_, 250 mM sucrose, 1 mM tryptophan, 0.1% (w/v) DDM, 250 μg ml^−1^ chloramphenicol and 0.1% EDTA-free complete proteinase inhibitors) and buffer D (50 mM HEPES pH 7.5/KOH, 250 mM KOAc, 25 mM Mg(OAc)_2_, 0.1% (w/v) DDM, 250 μg ml^−1^ chloramphenicol). Elution was performed with buffer D + 150 mM imidazole. The eluate was loaded on a sucrose gradient (buffer D + 10–40% (w/v) sucrose) and spun in a SW 40 Ti rotor (Beckman Coulter) at 54,322 × *g* for 16 h at 4 °C. The 70S peak was harvested and pelleted again in a TLA110 rotor (Beckman Coulter) at 434,513 × *g* for 2 h at 4 °C and resuspended in buffer D + 1 mM tryptophane. Purified RNCs were reconstituted with SRP, which was pre-incubated with either GMPP*N*P or pppGpp for 10 min at room temperature. A 10-fold molar excess of SRP was mixed with TnaC-stalled RNCs, incubated again for 10 min at room temperature and subsequently analyzed by cryo-EM.

### Cryo-EM sample preparation, data collection, and processing

A volume of 3.5 μL of the reconstituted SRP-RNC complex was applied to 2 nm pre-coated Quantifoil R3/3 holey carbon support grids and vitrified in liquid ethane using a Vitrobot mark IV (FEI Company, Netherlands) (wait time 45 s, blotting time 2 s). For the *B. subtilis* samples, 9976 and 9508 movies were collected on a Titan Krios at 300 kV for the pppGpp sample and the GMPP*N*P sample, respectively. The collection was recorded on a K2 Summit direct electron detector with an electron dose of approx. 1.06 e^−^/Å^2^ per frame for 10 frames (defocus range of 0.5 to 5 µm). The magnified pixel size was 1.059 Å/pixel. All frames were gain corrected and subsequently aligned and summed using MotionCor2^91^ and CTF parameters were determined using CTFFIND^92^ (version 4.1.13). After visual inspection of the micrographs, particles were picked using Gautomatch^93^ (version v0.56; http://www.mrc-lmb.cam.ac.uk/kzhang/). The particles were extracted and processed following the standard workflow in RELION 3.1^94^. For both datasets, the 2D classification was used to remove non-ribosomal particles and in the following 3D classification programmed 70S were selected. These were further sub-classified using spherical masks around the SRP Alu domain and the SRP S domain (Supplementary Fig. 7). SRP-bound RNCs were then refined and CTF-corrected. The particles were imported to Cryosparc v3.2.0^95^ and refined to a final resolution of 3.33 Å (pppGpp sample) and 2.96 Å (GMPP*N*P sample) (Supplementary Fig. 8).

For the *E. coli* samples, 14,285 and 15,042 movies were collected on a Titan Krios at 300 kV for the pppGpp sample and the GMPP*N*P sample, respectively. The collection was recorded on a Falcon II direct electron detector. The electron dose was approx. 2.5 e^−^/Å^2^ per frame for 16 frames (defocus range of 0.5 to 5 µm) and the magnified pixel size was 1.09 Å/pixel. All frames were corrected and aligned as described above. After visual inspection of the micrographs, crYOLO^96^ (version 1.7.6) was used for particle picking. For both datasets, the particles were extracted and processed following the standard workflow in RELION 3.1^94^. The 2D classification was used to remove non-ribosomal particles and in the following 3D classification programmed 70S were selected. First, a focused classification with a mask around SRP was used to enrich SRP-bound RNCs. Second, a focused classification with a mask around the NG domain of Ffh was used to classify for different conformations. No differences between the final classes of the two datasets could be observed (Supplementary Fig. 9). Therefore, in an alternative classification attempt, particles of both datasets were combined and sub-classified using spherical masks around SRP (Supplementary Fig. 10). The class containing the best density for the NG domain was selected and particles were separated according to the nucleotide dataset. SRP-bound RNCs were then refined and CTF-corrected to a final resolution of 3.1 Å (pppGpp sample) and 3.2 Å (GMPP*N*P sample) again without revealing differences for the NG domain conformation (Supplementary Fig. 11).

### Model building and refinement of cryo-EM data

Chimera version 1.13.1^97^ and ChimeraX version 1.1^98^ were used for rigid body fits and figures. The structures of *B. subtilis* ErmDL-stalled ribosome complex (rRNA and r-proteins; PDB 6HA1 https://www.rcsb.org/structure/6HA1)^99^, the *B. subtilis* MifM-stalled ribosome complex (mRNA, tRNA, nascent chain; PDB 3J9W https://www.rcsb.org/structure/3J9W)^52^ and of the *B. subtilis* signal recognition particle (SRP RNA, Ffh-M domain, signal sequence; PDB 4UE4 https://www.rcsb.org/structure/4UE4;^53^) were fitted into the cryo-EM map, side chains of proteins and rRNA were adjusted using Coot (version 0.8.9.2)^84^ and all models were real space refined using Phenix (version 1.19)^85^. The structure of the *B. subtilis* Ffh-NG domain bound to pppGpp (this study) was fitted into the cryo-EM-map and combined with the refined structure (Supplementary Table 2). The structure of the *E. coli* RNC in complex with SRP (PDB 5GAF https://www.rcsb.org/structure/5GAF^54^) was fitted into the obtained cryo-EM maps for interpretation and visualization. No further modeling of the *E. coli* RNCs with SRP was performed.

### Reporting summary

Further information on research design is available in the Nature Research Reporting Summary linked to this article.

## Supplementary information

Supplementary Information

Reporting Summary

## Data Availability

Coordinates and structure factors of the crystal structures and coordinates of the cryo-EM structure have been deposited at the Protein Data Bank with the accession codes: 7O9F, 7O9G, 7O9H, 7O9I, 7O5B. Cryo-EM maps have been deposited at the EMDB with the accession codes: 12734, 12735, 13839, 13840. All other data generated in this study are provided in the Supplementary Information and Source Data file, or are available from the corresponding authors upon request. Already published datasets used in this study are: 1OKK, 3NG1, 2YHS, 6HA1, 3J9W, 4UE4, 5GAF. Source data are provided with this paper.

## Source data

Source Data
