# Supplementary material for: Inhibition of SRP-dependent protein secretion by the bacterial alarmone (p)ppGpp
Source: Nat Commun. 2022 Feb 25;13:1069. doi: 10.1038/s41467-022-28675-0 (PMC8881573; doi:10.1038/s41467-022-28675-0)
Supplement: Supplementary file 1 — Supplementary Information [file 41467_2022_28675_MOESM1_ESM.pdf]

Supplementary Materials for:

Inhibition of SRP-dependent protein secretion by the bacterial alarmone (p)ppGpp

Laura Czech*[1,†], Christopher-Nils Mais[1,†], Hanna Kratzat[2], Pinku Sarmah[3,4], Pietro Giammarinaro[1], Sven-Andreas Freibert[5,6], Hanna Folke Esser[2], Joanna Musial[2], Otto Berninghausen[2], Wieland Steinchen[1], Roland Beckmann[2], Hans-Georg Koch[3], Gert Bange*[1,7]

[1]Center for Synthetic Microbiology (SYNMIKRO) and Department of Chemistry, Philipps-Universität Marburg; Marburg, Germany.

[2]Gene Center Munich, Department of Biochemistry, Ludwig-Maximilians-Universität, LMU; Munich, Germany.

[3]Institute of Biochemistry and Molecular Biology, Faculty of Medicine, Albert-Ludwigs-Universität Freiburg; Freiburg, Germany.

[4]Faculty of Biology, Albert-Ludwigs-Universität Freiburg; Freiburg, Germany.

[5]Institut für Zytobiologie, Philipps-Universität Marburg; Marburg, Germany.

[6]Core Facility 'Protein Biochemistry and Spectroscopy', Philipps-Universität Marburg; Marburg, Germany.

[7]Max-Planck Institute for terrestrial Microbiology; Marburg, Germany.

[†]These authors contribute equally to this work.

[*]Corresponding authors. Email: laura.czech@staff.uni-marburg.de, gert.bange@synmikro.uni-marburg.de

This PDF file includes:

Supplementary Figs. 1 to 11
Supplementary Tables S1 to S4

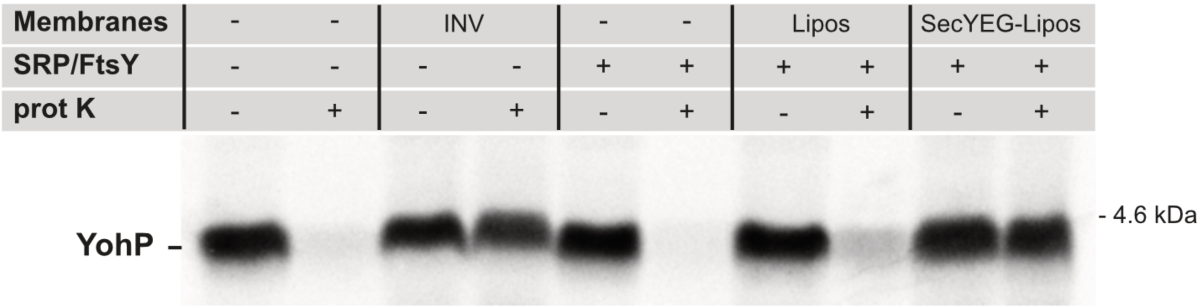

Supplementary Fig. 1. YohP membrane insertion requires SRP, FtsY and SecYEG. YohP was *in vitro* synthesized using a purified coupled transcription/translation system (CTF system) and translation was terminated by the addition of chloramphenicol (35 mg ml^{-1}). Samples were then centrifuged for removing ribosomes and aggregates; the supernatant was subsequently incubated with either INV-buffer, INV (inverted inner membrane vesicles), liposomes or SecYEG-proteoliposomes for 10 min in the presence of 10 μM GTP. When indicated, SRP and FtsY were present (20 ng μl^{-1}, each). Subsequently, one half of the sample was immediately TCA precipitated, while the other half was treated with proteinase K (prot K) before TCA precipitation. Samples were then separated by SDS-PAGE and analyzed by autoradiography. A representative gel of at least three independent experiments is shown.

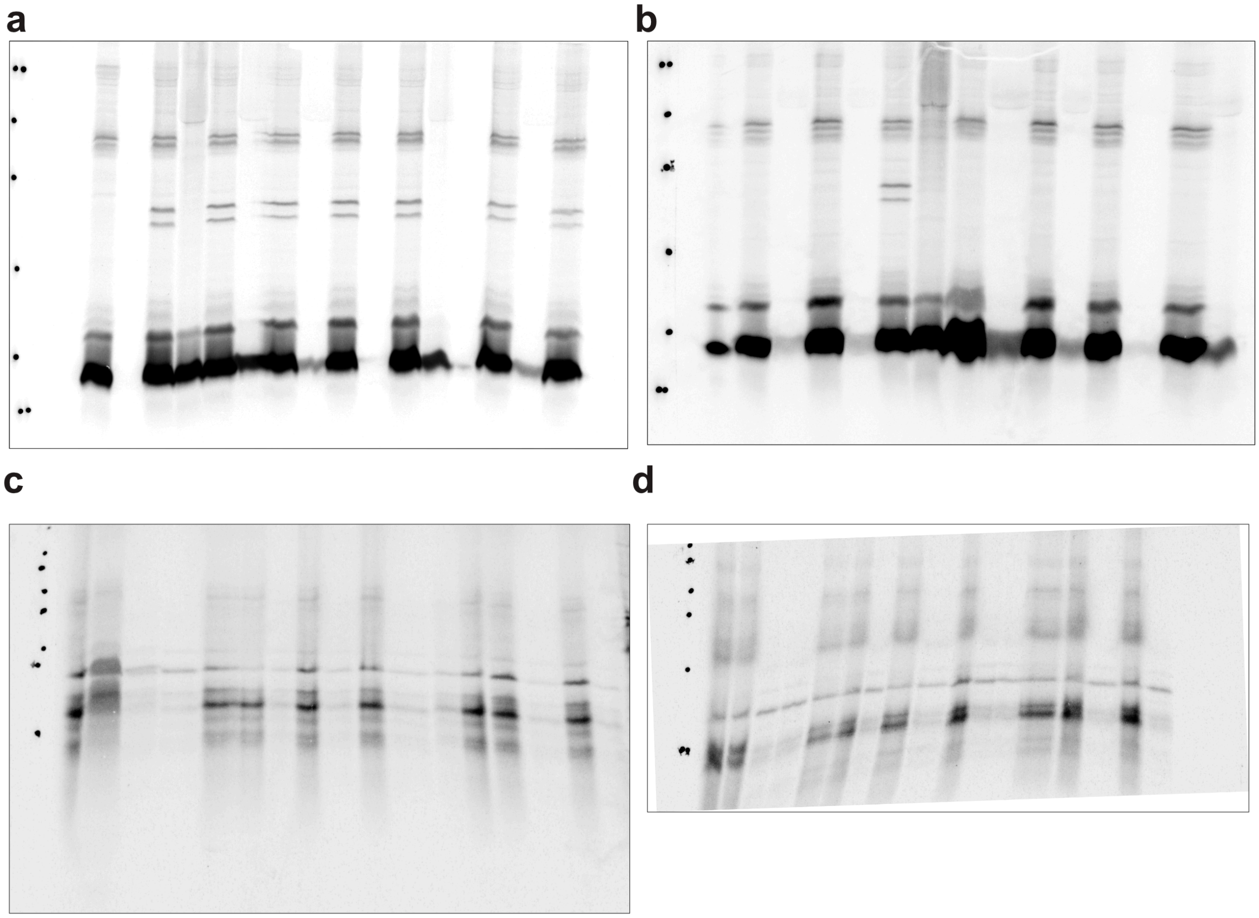

Supplementary Fig. 2 Uncropped images of main text Figure 1 and 2. (a) Uncropped image of Figure 1a. (b) Uncropped image of Figure 1c. (c) Uncropped image of Figure 2a. (d) Uncropped image of Figure 2b.

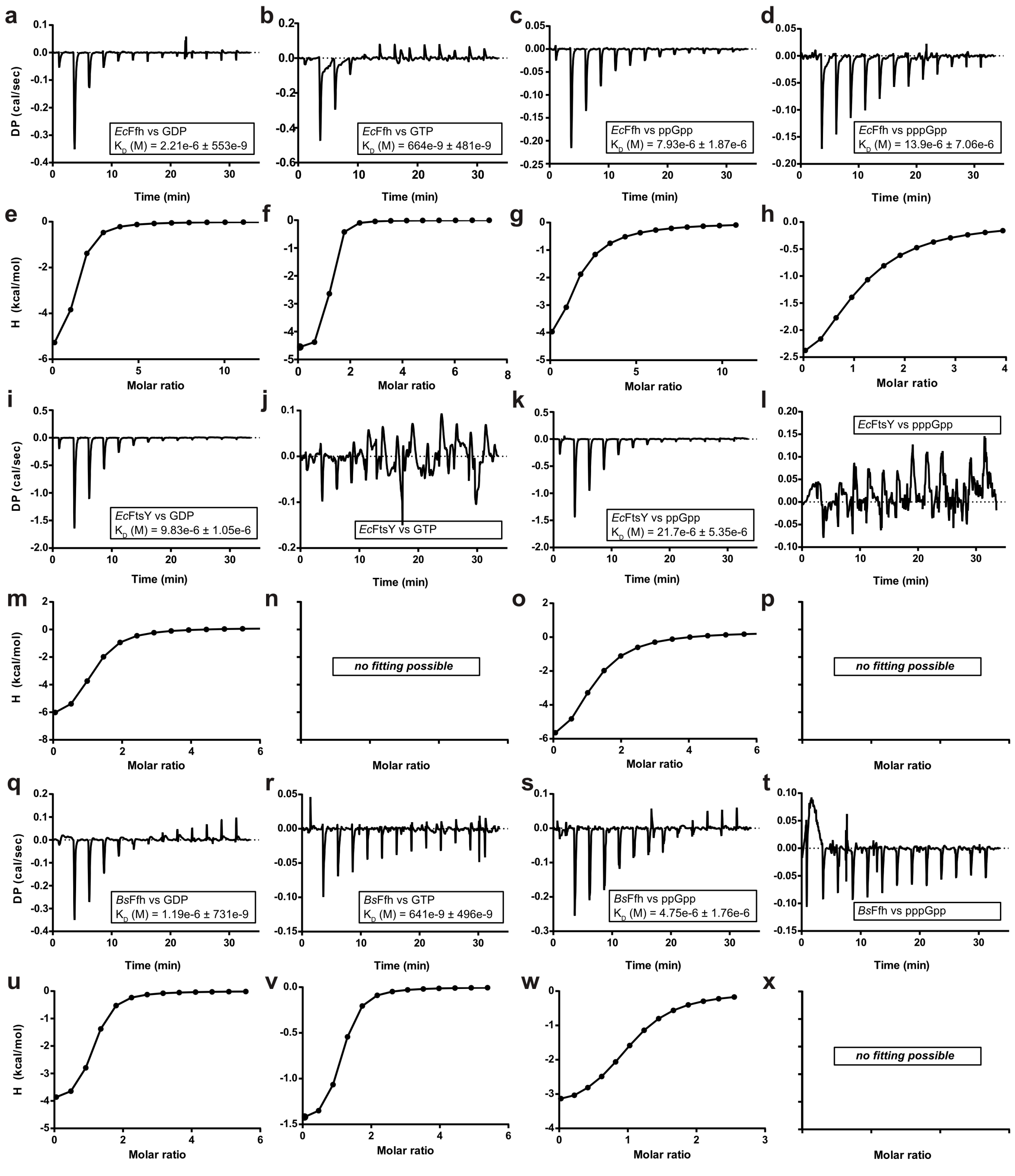

Supplementary Fig. 3. Ligand binding of Ffh and FtsY NG domains from *E. coli* and Ffh NG domain from *B. subtilis.* Purified NG domains of (*Ec*)Ffh (a-h), (*Ec*)FtsY (i-p) and (*Bs*)Ffh (q-x) were titrated with GDP, GTP, ppGpp and pppGpp and the binding was assessed by isothermal calorimetry, respectively. Panels a-d, i-l, and q-t show the thermograms for each ITC run, and panels e-h, m-p, and u-x are the resulting fitted plots given as heat versus ligand concentration. DP stands for differential power.

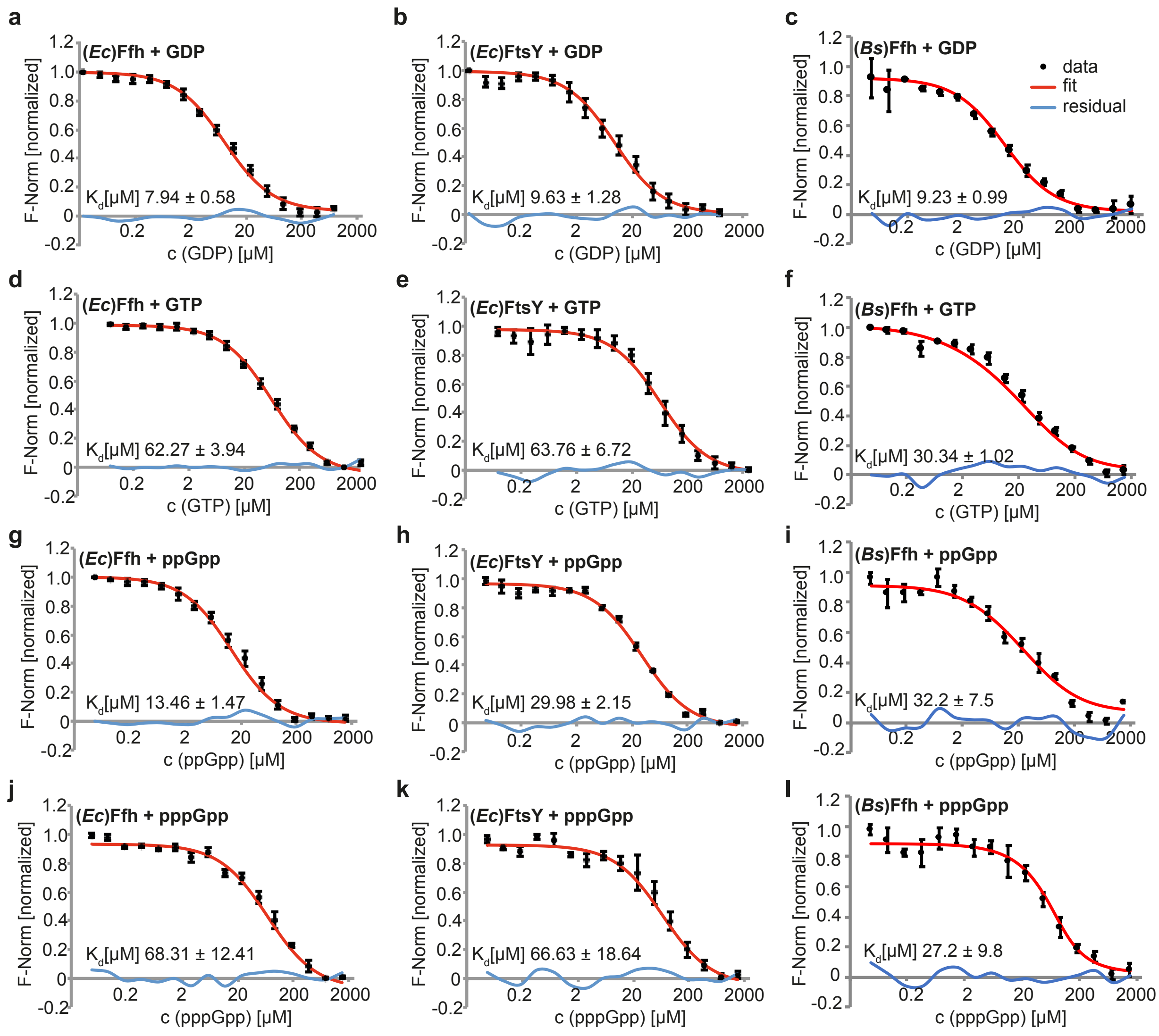

Supplementary Fig. 4. Ligand binding of Ffh and FtsY NG domains from *E. coli* and Ffh NG domain from *B. subtilis.* Purified NG domains of (*Ec*)Ffh, (*Ec*)FtsY and (*Bs*)Ffh were incubated with increasing concentrations of (a, b and c) GDP, (d, e and f) GTP, (g, h and i) ppGpp and (j, k and l) pppGpp and the binding was assessed by microscale thermophoresis, respectively. Data are presented as mean values +/- SD of n=2 biologically independent samples and each three technically independent measurements.

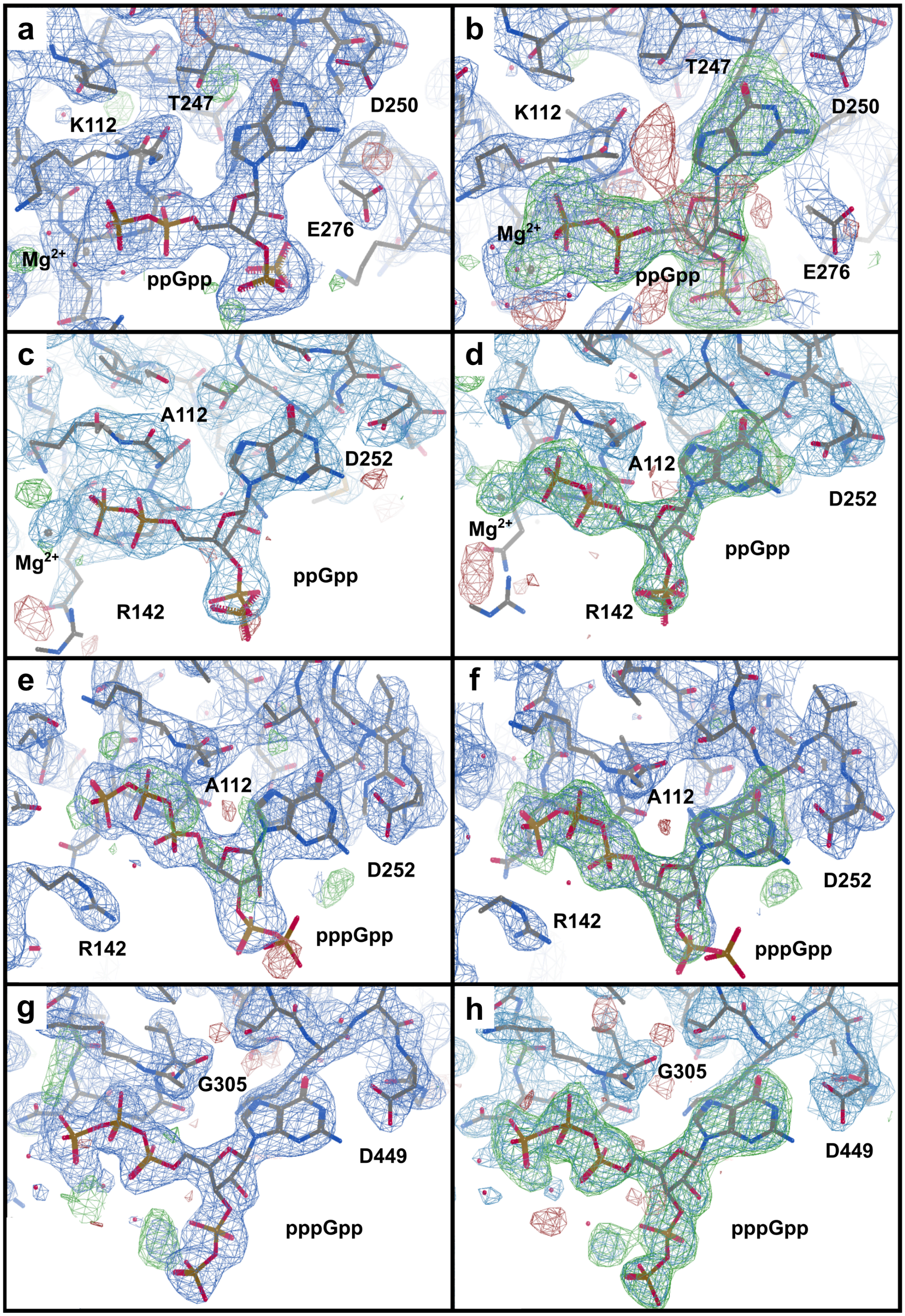

a
K112
T247
D250
E276
Mg2+
ppGpp
b
T247
K112
D250
Mg2+
ppGpp
E276
c
A112
D252
Mg2+
ppGpp
R142
d
A112
D252
Mg2+
ppGpp
R142
e
A112
D252
R142
pppGpp
f
A112
D252
R142
pppGpp
g
G305
D449
pppGpp
h
G305
D449
pppGpp

Supplementary Fig. 5. Substrate coordination in Ffh and FtsY. (a) The $2F_{obs}$-F_{calc} electron density after final refinement of (*Bs*)Ffh with ppGpp and Mg^{2+} is shown as a blue mesh at 1.5 σ. F_{obs}-F_{calc} difference electron density at 3 σ is shown as green mesh. Applies to all following densities. (b) Unbiased F_{obs}-F_{calc} difference electron density of (*Bs*)Ffh with ppGpp and Mg^{2+}. Bias was removed by refinement prior to incorporation of the ligand. Applies to all following unbiased densities. (c) Electron density after final refinement of (*Ec*)Ffh with ppGpp and Mg^{2+}. (d) Unbiased electron density of (*Ec*)Ffh with ppGpp and Mg^{2+}. (e) Electron density after final refinement of (*Ec*)Ffh with pppGpp. (f) Unbiased electron density of (*Ec*)Ffh with pppGpp. (g) Electron density after final refinement of (*Ec*)FtsY with pppGpp. (h) Unbiased electron density of (*Ec*)FtsY with pppGpp.

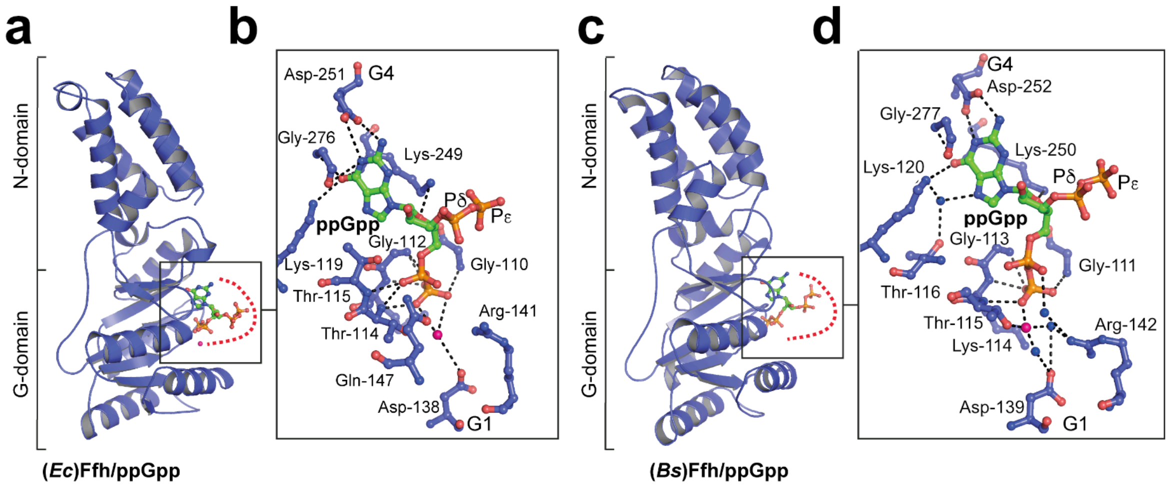

Supplementary Fig. 6. Overall fold and zoom into the nucleotide binding side of the NG domains of (*Ec*)Ffh and (*Bs*)Ffh both in complex with ppGpp and Mg^{2+}. (a and c) Overall fold of the NG domain of (*Ec*)Ffh (a) and (*Bs*)Ffh (c). (b and d) Coordination of the alarmone ppGpp (green) and Mg^{2+} (pink) within the nucleotide binding side of (*Ec*)Ffh (c) and (*Bs*)Ffh (d). Waters are shown in blue.

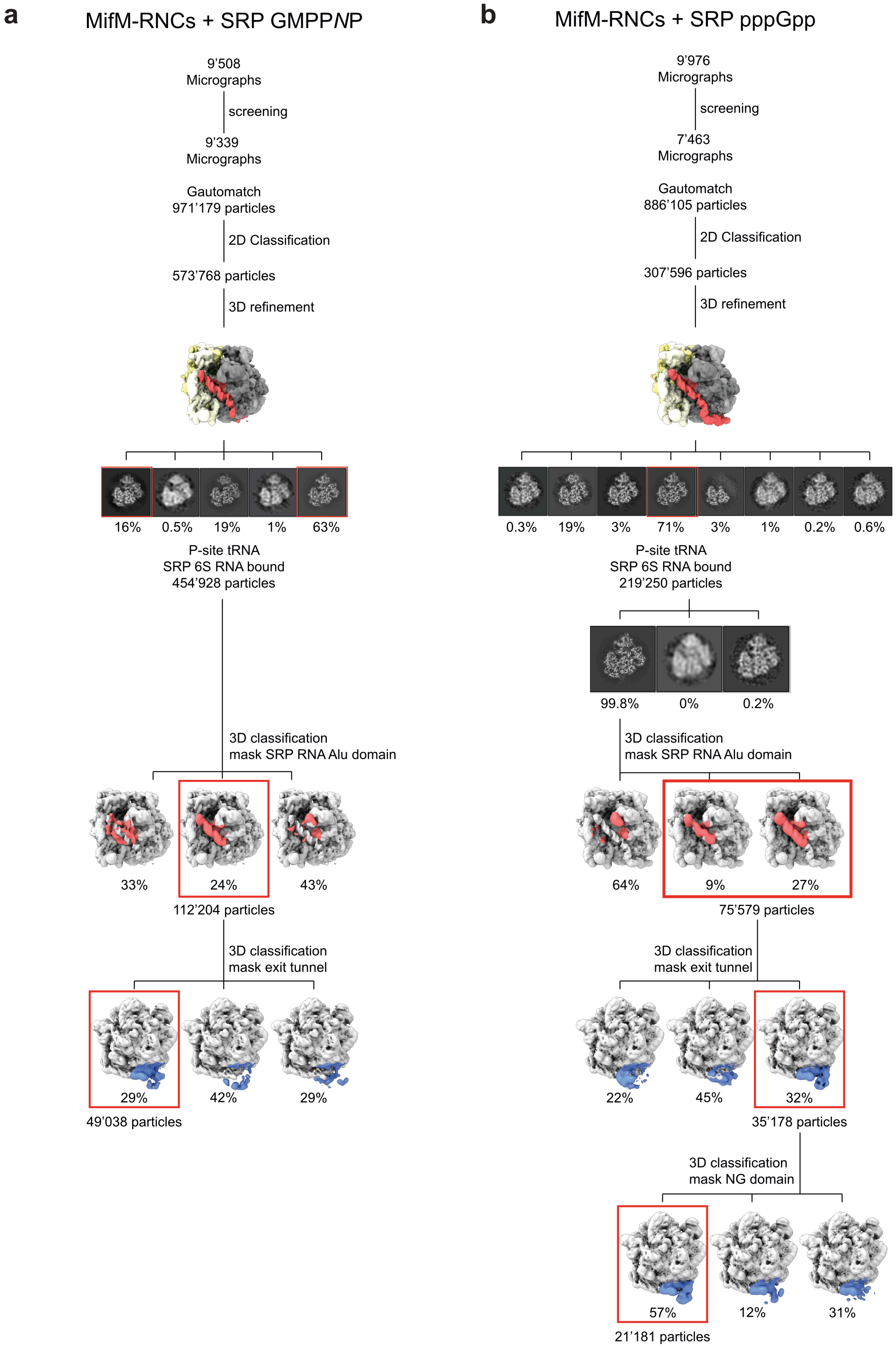

Supplementary Fig. 7. Sorting schemes of the *B. subtilis* cryo-EM datasets. Sorting scheme of the dataset of (*Bs*)SRP-bound MifM-stalled RNCs preincubated with GMPP*N*P (a) and RNCs preincubated with pppGpp (b). In the GPMP*N*P data set, classification attempts to sort for stable conformations of the NG domain of Ffh were unsuccessful.

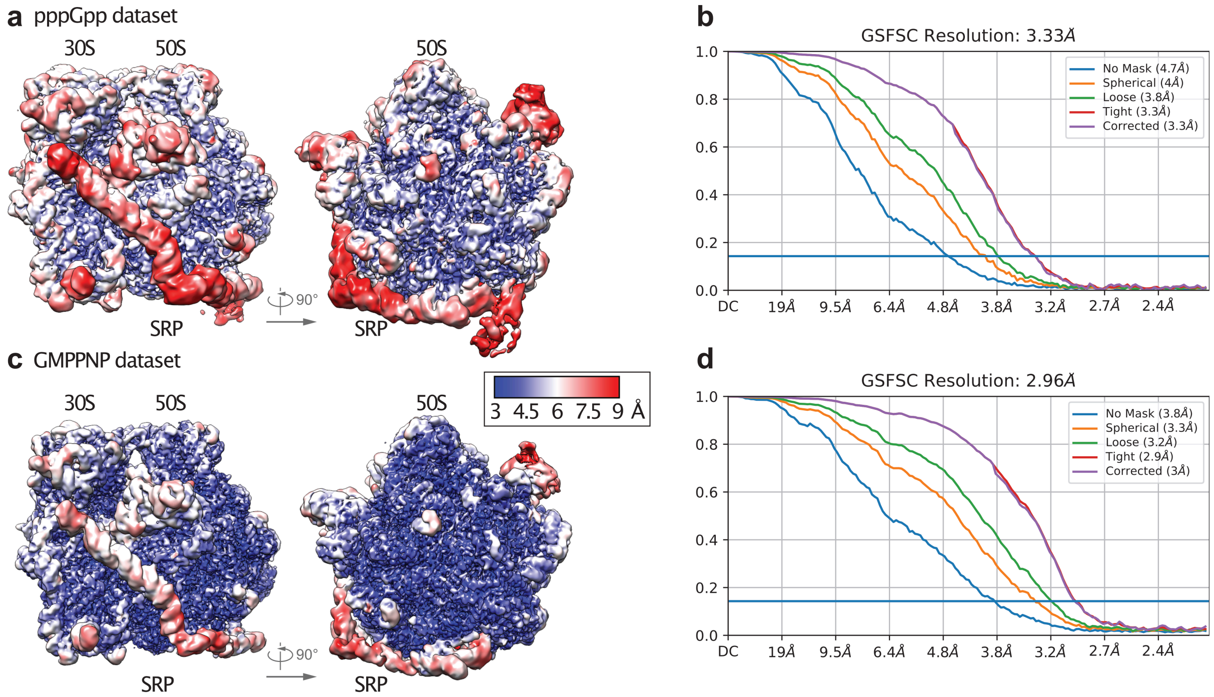

Supplementary Fig. 8. Resolution of the SRP-RNC complexes. pppGpp dataset cryo-EM map of (*Bs*)SRP-bound MifM-stalled RNCs filtered and colored at local resolution (a) and the corresponding Gold standard Fourier Shell Correlation (GSFSC) curve from Cryosparc (b). GMPP*N*P dataset cryo-EM map of (*Bs*)SRP-bound MifM-stalled RNCs filtered and colored at local resolution (c) and the corresponding GSFSC curve from Cryosparc (d).

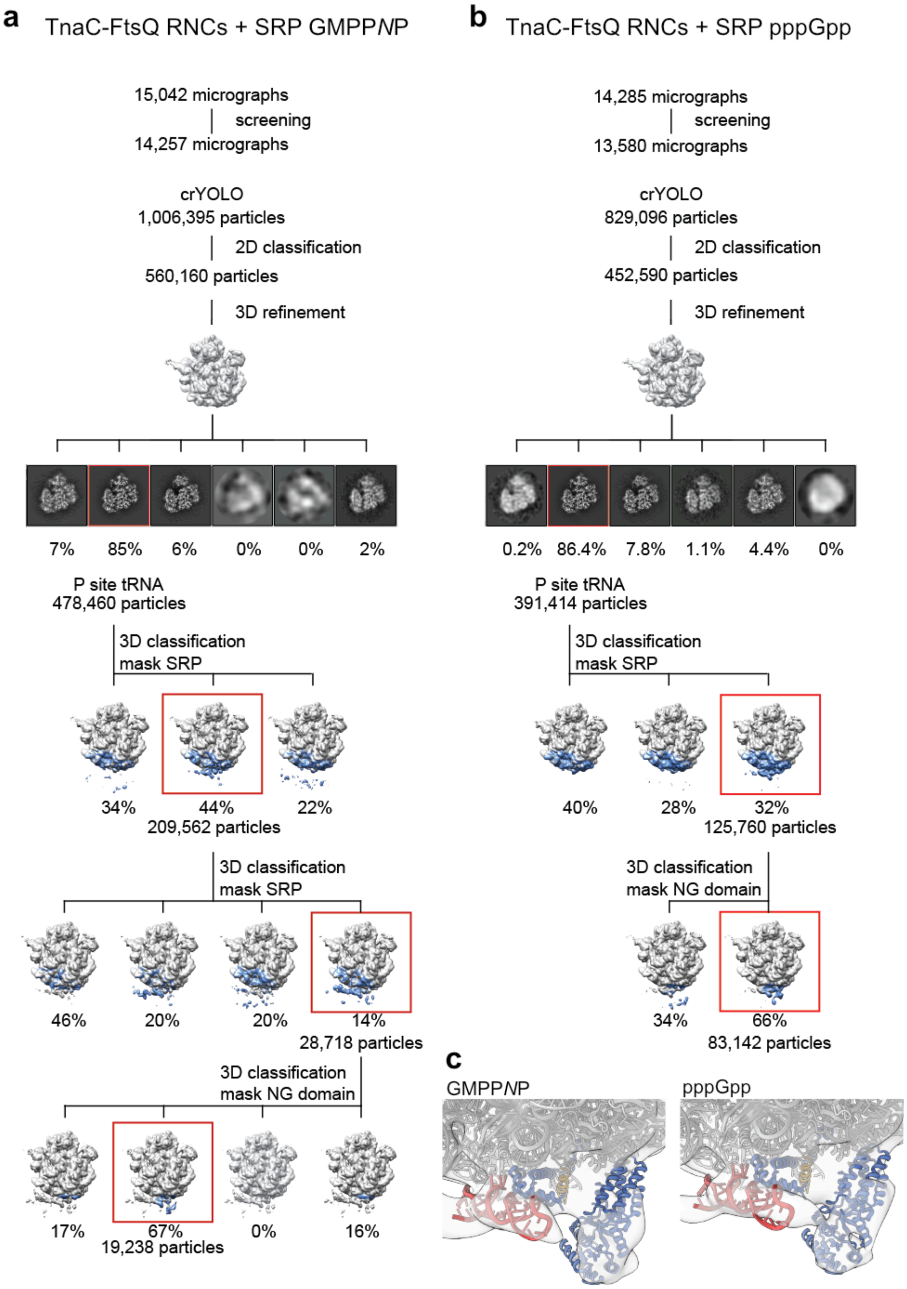

Supplementary Fig. 9. Sorting scheme of the jointly processed *E. coli* cryo-EM datasets. Initial processing of the datasets of (*Ec*)SRP-bound TnaC-stalled RNCs preincubated with GMPP*N*P (a, left) and RNCs preincubated with pppGpp (b, right). To facilitate an unbiased comparison, the data sets were combined to sort for different conformations of the NG domain of SRP. Afterwards the particles were separated and refined individually. (c) Comparison of a detailed view of SRP bound to *E. coli* RNCs in complex with GMPP*N*P and pppGpp.

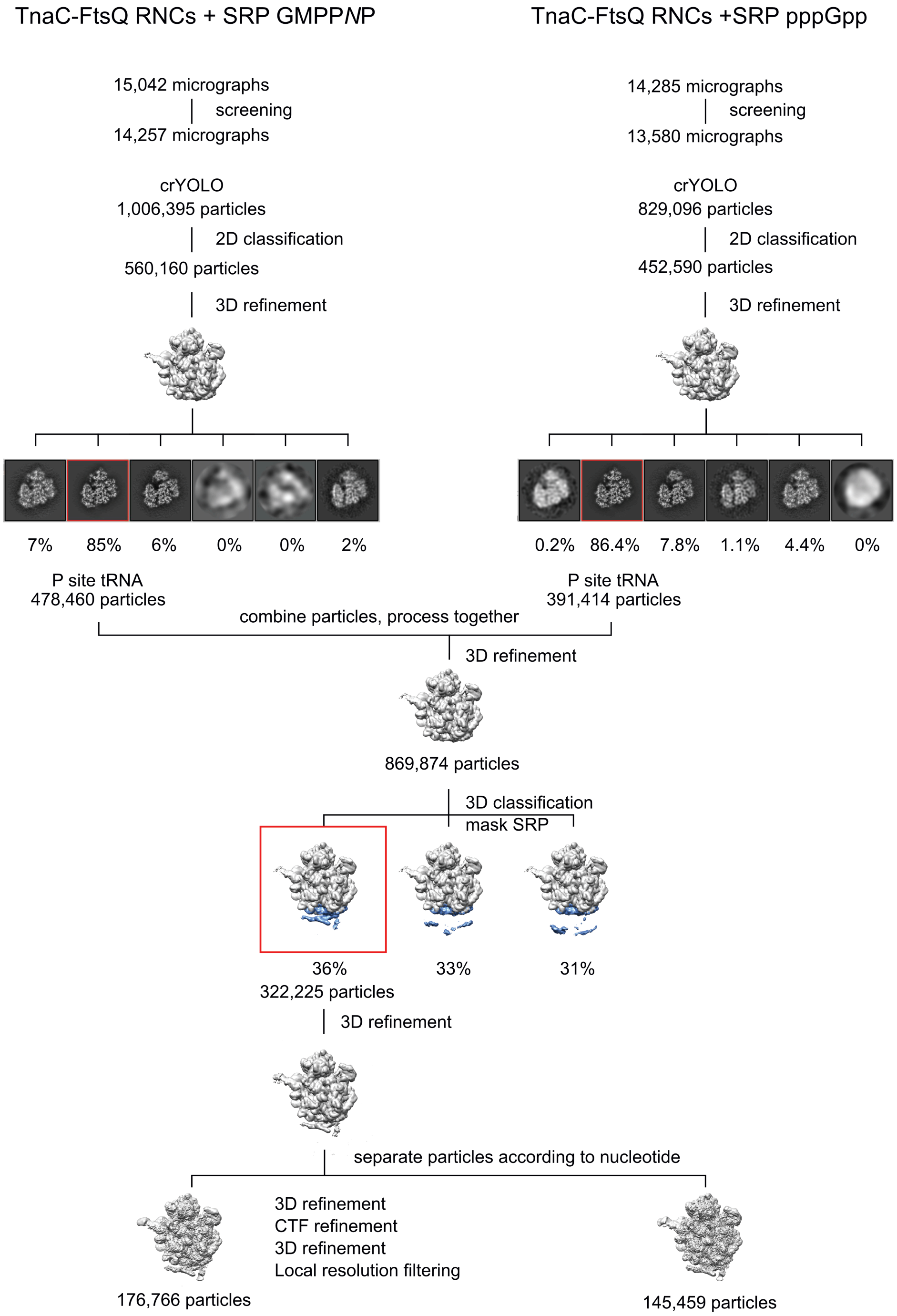

Supplementary Fig. 10. Sorting schemes of the *E. coli* cryo-EM datasets. Sorting scheme of the datasets of (*Ec*)SRP-bound TnaC-stalled RNCs preincubated with GMPP*N*P (left) and RNCs preincubated with pppGpp (right). To avoid differences based on sorting strategies and to facilitate an unbiased comparison, the data sets were combined to sort for different conformations of the NG domain of SRP. Afterwards the particles were separated and refined individually.

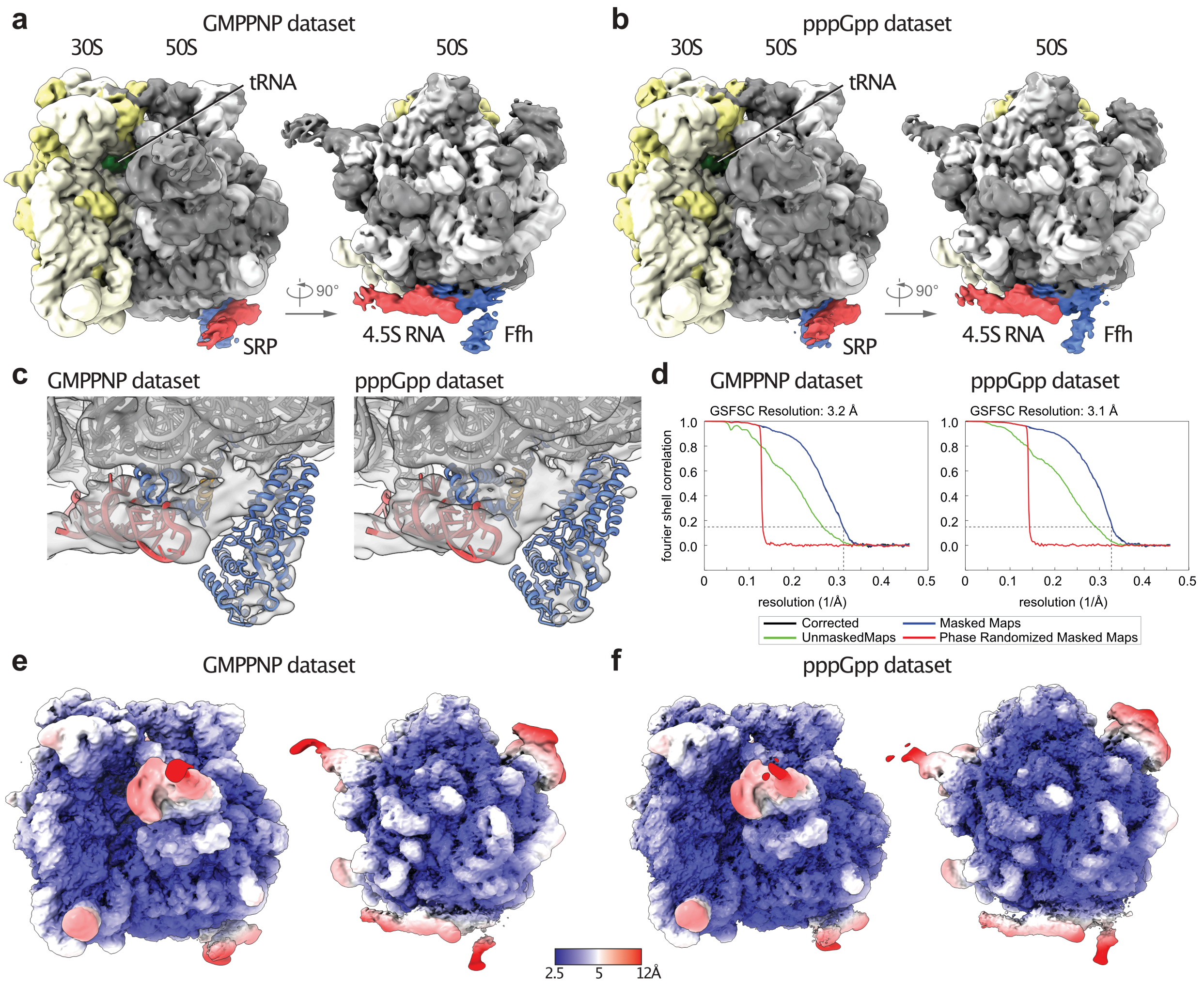

Supplementary Fig. 11. Cryo-EM structures of *E. coli* SRP-RNC complexes. GMPP*N*P dataset cryo-EM map (a) and pppGpp dataset cryo-EM map (b) of (*Ec*)SRP-bound TnaC-stalled RNCs filtered at 8Å with 30S small ribosomal subunit in yellow, 50S large ribosomal subunit in grey, and SRP in red (4.5*S* RNA) and blue (Ffh). (c) Comparison of Ffh-NG domain cryo-EM densities for the GMPP*N*P and pppGpp dataset; the fitted model is (*Ec*)SRP-bound RNCs with GMPP*N*P (PDB 5GAF), which was aligned on the 50S. (d) Gold standard Fourier Shell Correlation (GSFSC) curves of the two final volumes from Relion. (e) GMPP*N*P dataset cryo-EM map of (*Ec*)SRP-bound TnaC-stalled RNCs filtered and colored at local resolution. (f) pppGpp dataset cryo-EM map of (*Ec*)SRP-bound TnaC-stalled RNCs filtered and colored at local resolution.

Supplementary Table 1. Data collection and refinement statistics.

	(*Ec*)Ffh, pppGpp	(*Ec*)FtsY, pppGpp	(*Ec*)Ffh, ppGpp+Mg^{2+}	(*Bs*)Ffh, ppGpp+Mg^{2+}
**Data collection**				
Space group	$P4_1$	$P2_12_12_1$	$P12_11$	$P4_12_12$
Cell dimensions				
a, *b*, *c* (Å)	58.06<br>58.06<br>101.68	74.48<br>90.71<br>106.64	48.63<br>38.2<br>78.44	163.28<br>163.28<br>93.47
α, β, γ (°)	90<br>90<br>90	90<br>90<br>90	90<br>96.273<br>90	90<br>90<br>90
Wavelength (Å)	0.976253	0.976253	0.97626	0.976253
Resolution (Å)	38.25 - 2.492<br>(2.581 - 2.492)	43.36 - 2.4<br>(2.486 - 2.4)	43.26 - 2.803<br>(2.642 - 2.551)	49.12 - 2.511<br>(2.601 - 2.511)
R_{merge}	0.0769 (0.4526)	0.1867 (1.1)	0.02444 (0.0704)	0.1376 (3.609)
$I / \sigma I$	25.77 (4.74)	8.85 (2.81)	32.05 (12.07)	17.62 (0.99)
Completeness (%)	99.40 (95.94)	90.78 (99.79)	97.41 (96.54)	99.90 (99.28)
Redundancy	19.2 (14.4)	6.2 (6.6)	3.4 (3.4)	25.7 (26.2)
CC1/2	0.999 (0.969)	0.993 (0.782)	0.999 (0.996)	0.999 (0.488)
**Refinement**				
Resolution (Å)	38.25 - 2.492	43.36 - 2.4	43.26 - 2.803	49.12 - 2.511
No. reflections	11746 (1115)	26244 (2818)	7078 (697)	43593 (4268)
R_{work} / R_{free}	0.24/0.27	0.21/0.26	0.21/0.24	0.22/0.26
No. atoms	2206	4809	2283	6930
Protein	2152	4642	2237	6803
Ligand/ion	40	80	37	111
Water	14	87	9	16
B-factors	64.34	44.91	60.14	101.23
Protein	64.15	44.76	60.22	101.48
Ligand/ion	76.60	55.57	60.59	91.28
Water	58.18	43.17	38.63	65.86
R.m.s. deviations				
Bond lengths (Å)	0.009	0.010	0.011	0.008
Bond angles (°)	1.44	1.30	1.56	1.22
Ramachandran				
Favored (%)	97.18	98.67	96.92	97.73
Allowed (%)	2.46	1.33	3.08	2.27
Outliers (%)	0.35	0.00	0.00	0.00

*Values in parentheses are for highest-resolution shell.

Data were collected on ID30A-3 (MASSIF-3, ESRF) and P14 (DESY).

Supplementary Table 2. Data collection and refinement cryo-EM *B. subtilis* (*Bs*) datasets.

	(*Bs*) SRP-RNC (pppGpp) PDB 7O5B EMDB 12734	(*Bs*) SRP-RNC (GMPP*N*P) EMDB 12735
**Data collection**		
Voltage (kV)	300	
Electron exposure (e–/$Å^2$)	25	
Defocus range (µm)	−0.5 to −5	
Pixel size (Å)	1.059	
Symmetry imposed	C1	
**Refinement**		
Particle images (no.)	21,229	49,287
Map resolution (Å)	3.33	2.96
FSC threshold	0.143	0.143
Map sharpening B factor ($Å^2$)	-85.7	-73.5
**Model composition**		
Correlation coefficient (%; Phenix)	0.80	
Models used (PDB codes)	6HA1, 3JW9, 4UE4	
Non-hydrogen atoms	150,422	
Protein residues	6,001	
RNA bases	4,883	
**R.m.s. deviations**		
Bond lengths (Å)	0.006	
Bond angles (°)	0.978	
**Validation**		
MolProbity score	1.33	
Clash score	5.11	
Rotamer outliers (%)	0.15	
**Ramachandran plot**		
Favored (%)	97.74	
Allowed (%)	2.1	
Disallowed (%)	0.15	
**Validation RNA**		
Correct sugar pucker (%)	99.18	
Good backbone conf. (%)	72.31	

Supplementary Table 3. Data collection and refinement cryo-EM *E. coli* (*Ec*) datasets.

	**(*Ec*) SRP-RNC (pppGpp)**<br>**EMDB 13839**	**(*Ec*) SRP-RNC (GMPP*N*P)**<br>**EMDB 13840**
**Data collection**		
Voltage (kV)	300	
Electron exposure (e–/Å^2)	40	
Defocus range (μm)	−0.5 to −4	
Pixel size (Å)	1.09	
Symmetry imposed	C1	
**Refinement**		
Particle images (no.)	145,459	176,766
Map resolution (Å)	3.1	3.2
FSC threshold	0.143	0.143
Map sharpening B factor (Å^2)	-30	-30

Supplementary Table 4. Plasmids and primers used in this study.

Plasmid	Primer name	sequence
**pNM101**	**EcFfh fwd**	TTAACCATGGGCTTTGATAATTTAACCGATCGTTTGTCG
(pET24d_EcFfh-NG)	**EcFfh rev 6H**	TTAAACTCGAGTTTTAGATGGTGATGGTGATGGTGGCCGTCACCTTTTTTCAGC
**pNM103**	**EcFtsY fwd**	TTAACCATGGGCGGCCATCACCATCACCA
(pET24d_EcFtsY-NG)	**EcFtsY rev 6H**	TTAAACTCGAGTTTTAGATGGTGATGGTGATGGTGTCCTCTCGGGCAAAAAG
**pLC163**	**BsFfh fwd**	TTAAGGTCTCCCATGGGCTTTGAAGGATTAGCCGACCGACTGC
(pET24d_BsFfh-NG)	**BsFfh rev**	TTAAGGTCTCCTCGAGGTCGCCCATGCCGAGAATCC
**pLC164**	**BsFtsY fwd**	TTAAGGTCTCCCATGGGCAGCTTTTTTAAAAAATTAAAAGAGAAAATCACAAAACAG
(pET24d_BsFtsY-NG)	**BsFtsY rev**	TTAAGGTCTCCTCGAGATCGTCGGCTTTTTCCACTAAATC
