# Supplementary material for: Inhibition of SRP-dependent protein secretion by the bacterial alarmone (p)ppGpp
Source: Nat Commun. 2022 Feb 25;13:1069. doi: 10.1038/s41467-022-28675-0 (PMC8881573; doi:10.1038/s41467-022-28675-0)
Supplement: Supplementary file 2 — Reporting Summary [file 41467_2022_28675_MOESM2_ESM.pdf]

nature research

Corresponding author(s): Czech, Laura
Bange, Gert

Last updated by author(s): May 21, 2021

Reporting Summary

Nature Research wishes to improve the reproducibility of the work that we publish. This form provides structure for consistency and transparency in reporting. For further information on Nature Research policies, see our Editorial Policies and the Editorial Policy Checklist.

Statistics

For all statistical analyses, confirm that the following items are present in the figure legend, table legend, main text, or Methods section.

n/a	Confirmed	
☐	☒	The exact sample size (*n*) for each experimental group/condition, given as a discrete number and unit of measurement
☐	☒	A statement on whether measurements were taken from distinct samples or whether the same sample was measured repeatedly
☐	☒	The statistical test(s) used AND whether they are one- or two-sided<br>*Only common tests should be described solely by name; describe more complex techniques in the Methods section.*
☒	☐	A description of all covariates tested
☒	☐	A description of any assumptions or corrections, such as tests of normality and adjustment for multiple comparisons
☐	☒	A full description of the statistical parameters including central tendency (e.g. means) or other basic estimates (e.g. regression coefficient) AND variation (e.g. standard deviation) or associated estimates of uncertainty (e.g. confidence intervals)
☒	☐	For null hypothesis testing, the test statistic (e.g. *F*, *t*, *r*) with confidence intervals, effect sizes, degrees of freedom and *P* value noted<br>*Give P values as exact values whenever suitable.*
☒	☐	For Bayesian analysis, information on the choice of priors and Markov chain Monte Carlo settings
☒	☐	For hierarchical and complex designs, identification of the appropriate level for tests and full reporting of outcomes
☒	☐	Estimates of effect sizes (e.g. Cohen's *d*, Pearson's *r*), indicating how they were calculated

Our web collection on statistics for biologists contains articles on many of the points above.

Software and code

Policy information about availability of computer code

Data collection	Äkta system: UNICORN 7 (Cytiva); ITC: MicroCal PEAQ-ITC (©Malvern Panalytical); MST system: NanoTemper Control Version 2.0.2.29 (NanoTemper); X-Ray Data Collection: MxCube3, MxCube2 (https://github.com/mxcube); Cryo-EM: relion's 3.1 implementation of MotionCor2, CTFFIND 4.1.13, Gautomatch v0.56 (http://www.mrc-lmb.cam.ac.uk/kzhang/), RELION 3.1
Data analysis	Microsoft Excel for Mac 2011 Version 14.6.8; GraphPad Prism 5 for Mac OS X; ImageQuant (GE Healthcare); ITC: MicroCal PEAQ-ITC Analysis Software (©Malvern Panalytical); MST system: NanoTemper Analysis Version 1.5.37 and 1.2.009 (NanoTemper); Origin8G (OriginLab); Pymol Version 1.8.2.0 (Schroedinger); Coot Version 0.8.9.2 and 0.9.4.1 (doi:10.1107/S0907444910007493); Phenix Version 1.17.1-3660 and 1.19 (https://doi.org/10.1107/S2059798319011471); XDS Version January 31, 2020; Cryosparc v3.2.0; Chimera Version 1.13.1, ChimeraX version 1.1; crYOLO version 1.7.6

For manuscripts utilizing custom algorithms or software that are central to the research but not yet described in published literature, software must be made available to editors and reviewers. We strongly encourage code deposition in a community repository (e.g. GitHub). See the Nature Research guidelines for submitting code & software for further information.

Data

Policy information about availability of data

All manuscripts must include a data availability statement. This statement should provide the following information, where applicable:

- Accession codes, unique identifiers, or web links for publicly available datasets
- A list of figures that have associated raw data
- A description of any restrictions on data availability

Data availability. Coordinates and structure factors of the crystal structures and coordinates of the cryo-EM structure have been deposited at the Protein Data Bank with the accession codes: 7O9F (https://doi.org/10.2210/pdb7O9F/pdb), 7O9G (https://doi.org/10.2210/pdb7O9G/pdb), 7O9H (https://doi.org/10.2210/pdb7O9H/

pdb), 7O9I (https://doi.org/10.2210/pdb7O9I/pdb), 7O5B (https://www.rcsb.org/structure/7O5B). Cryo EM maps have been deposited at the EMDB with the accession codes: 12734 (https://www.emdataresource.org/EMD-12734), 12735 (https://www.emdataresource.org/EMD-12735), 13839 (https://www.emdataresource.org/EMD-13839), 13840 (https://www.emdataresource.org/EMD-13840). All other data generated in this study are provided in the Supplementary Information/Source Data file or are available upon request. Requests for materials and correspondence should be addressed to LC and GB. Already published datasets used in this study are: 1OKK (https://www.rcsb.org/structure/1OKK), 3NG1 (https://www.rcsb.org/structure/3NG1), 2YHS (https://www.rcsb.org/structure/2YHS), 6HA1 (https://www.rcsb.org/structure/6HA1), 3J9W (https://www.rcsb.org/structure/3J9W), 4UE4 (https://www.rcsb.org/structure/4UE4), 5GAF (https://www.rcsb.org/structure/5GAF).

Field-specific reporting

Please select the one below that is the best fit for your research. If you are not sure, read the appropriate sections before making your selection.

- [x] Life sciences
- [ ] Behavioural & social sciences
- [ ] Ecological, evolutionary & environmental sciences

For a reference copy of the document with all sections, see nature.com/documents/nr-reporting-summary-flat.pdf

Life sciences study design

All studies must disclose on these points even when the disclosure is negative.

Sample size	Experiments were performed in (at least) two (biological) replicates (see also: replication).
Data exclusions	We did not exclude any data from consideration.
Replication	Fig. 1 and 2: Membrane insertion and floatation analyses have been performed in at least n=3 independent experiments. In case of LepB (Fig. 2) n=4 independent experiments were performed.<br>Fig. 3 c and d: GTPase data represent mean values (± SD) of n=3 replicates.<br>Fig.3 e: ITC measurments have been performed at least twice and MST data are presented as mean values +/- SD of n=2 biologically independent samples and each three technically independent measurements.
Randomization	No randomization was necessary for this study because investigators were comparing well studied specific proteins under well controlled conditions (e.g. addition of different nucleotides). No human or animal subjects were used in the study. Randomization is not generally used in this field.
Blinding	Investigators were not blinded. Blinding during collection was not needed because conditions were well controlled. Blinding is also not necessary because the results are quantitative and did not require subjective judgment or interpretation. Blinding is not typically used in the field. NOT APPLICABLE

Reporting for specific materials, systems and methods

We require information from authors about some types of materials, experimental systems and methods used in many studies. Here, indicate whether each material, system or method listed is relevant to your study. If you are not sure if a list item applies to your research, read the appropriate section before selecting a response.

Materials & experimental systems

n/a	Involved in the study	
☒	☐	Antibodies
☒	☐	Eukaryotic cell lines
☒	☐	Palaeontology and archaeology
☒	☐	Animals and other organisms
☒	☐	Human research participants
☒	☐	Clinical data
☒	☐	Dual use research of concern

Methods

n/a	Involved in the study	
☒	☐	ChIP-seq
☒	☐	Flow cytometry
☒	☐	MRI-based neuroimaging
